# Chemometric Analysis Evidencing the Variability in the Composition of Essential Oils in 10 Salvia Species from Different Taxonomic Sections or Phylogenetic Clades

**DOI:** 10.3390/molecules29071547

**Published:** 2024-03-29

**Authors:** Ekaterina-Michaela Tomou, Panagiota Fraskou, Konstantina Dimakopoulou, Eleftherios Dariotis, Nikos Krigas, Helen Skaltsa

**Affiliations:** 1 Department of Pharmacognosy & Chemistry of Natural Products, Faculty of Pharmacy, National and Kapodistrian University of Athens, Panepistimiopolis, Zografou, 15771 Athens, Greece; pfraskou@pharm.uoa.gr; 2 Department of Hygiene, Epidemiology and Medical Statistics, Medical School, National and Kapodistrian University of Athens, 11527 Athens, Greece; kdimakop@med.uoa.gr; 3 Institute of Plant Breeding and Genetic Resources, Hellenic Agricultural Organization DEMETER (ELGO Dimitra), 57001 Thermi, Greecenkrigas@elgo.gr (N.K.)

**Keywords:** sage, volatile compounds, GC–MS, chemotaxonomy, medicinal–aromatic plants, MAPs, review, yield

## Abstract

Essential oil (EO) of *Salvia* spp. has been widely used for culinary purposes and in perfumery and cosmetics, as well as having beneficial effects on human health. The present study aimed to investigate the quantitative and qualitative variations in EOs in wild-growing and cultivated pairs of samples from members in four *Salvia* sections or three clades, namely *S. argentea* L. (Sect. *Aethiopis*; Clade I-C), *S. ringens* Sm. (Sect. *Eusphace*; Clade I-D), *S. verticillata* L. (Sect. *Hemisphace*; Clade I-B), *S. amplexicaulis* Lam., and *S. pratensis* L. (Sect. *Plethiosphace*; Clade I-C). Furthermore, the natural variability in EO composition due to different genotypes adapted in different geographical and environmental conditions was examined by employing members of three *Salvia* sections or two phylogenetic clades, namely *S. sclarea* L. (six samples; Sect. *Aethiopis* or Clade I-C), *S. ringens* (three samples; Sect. *Eusphace* or Clade I-D), and *S. amplexicaulis* (five samples; Sect. *Plethiosphace* or Clade I-C). We also investigated the EO composition of four wild-growing species of two *Salvia* sections, i.e., *S. aethiopis* L., *S. candidissima* Vahl, and *S. teddii* of Sect. *Aethiopis*, as well as the cultivated material of *S. virgata* Jacq. (Sect. *Plethiosphace*), all belonging to Clade I-C. The EO composition of the Greek endemic *S. teddii* is presented herein only for the first time. Taken together, the findings of previous studies are summarized and critically discussed with the obtained results. Chemometric analysis (PCA, HCA, and clustered heat map) was used to identify the sample relationships based on their chemical classes, resulting in the classification of two distinct groups. These can be further explored in assistance of classical or modern taxonomic *Salvia* studies.

## 1. Introduction

The genus *Salvia* L. is the largest genus of the Lamiaceae family [1], with approximately 1000 accepted species to date [2], and they are distributed in the temperate, subtropical, and tropical regions from both the Old and New World, extending from Central and South America (above 500 species) to Central Asia and the Mediterranean Region (above 250 species) and East Asia (above 90 species) [3]. Taxonomically, the genus *Salvia* has continuously raised conflicting opinions regarding its classification [1]. The genus *Salvia* has been traditionally divided into four morphologically circumscribed subgenera (Subgen. *Salvia*, *Sclarea*, *Calosphace*, and *Leonia*), which include 12 sections, comprising members of Sect. *Hymenosphace*, *Eusphace*, and *Drymosphace*, as well as members of Sect. *Horminum*, *Aethiposis*, and *Plethiosphace*, which occur in the Old World, while members of Sect. *Calosphace* occur in the New World, and members of Sect. *Echinosphace*, *Pycnosphace*, *Heterosphace*, *Notiosphace*, and *Hemisphace* occur in both of them [4]. Phylogenetically, the genus *Salvia* has also been disputed as being monophyletic or polyphyletic [3] or paraphyletic [5]. These modern studies have re-circumscribed *Salvia* to consist of three separate clades (*Salvia* clades I–III), each with different sister groups [3], or they have embedded five other small genera (*Dorystaechas*, *Meriandra*, *Perovskia*, *Rosmarinus*, and *Zhumeria*) in a broadly defined *Salvia* circumscription [5]. In Greece, 26 *Salvia* taxa (species and subspecies) are found in different geographical areas [6], some of which are single-country endemics such as *S. eichleriana* Heldr. ex Halácsy and *S. teddii* Turrill.

Some members of the genus *Salvia* have great economic importance such as *S. officinalis* L., *S. fruticosa* Mill. (trilobed sage; Greek sage), *S. lavandulifolia* Vahl. (Spanish sage), *S. verbenaca* L., *S. sclarea* L. (clary sage), and *S. tomentosa* Mill. [7,8]. It is estimated that the volume production of essential oil (EO) of *S. sclarea*, *S. officinalis*, and *S. lavandulifolia* ranged between 50 and 100 tonnes per year [7].

With a genus name alluding to strong medicinal power and effective therapeutic properties (*Salvia* originated from the Latin word “salvare” meaning “to heal”) [9] for human health, several *Salvia* spp. have been used worldwide in traditional medicine since ancient times to treat various diseases like digestive disorders, inflammations/infections of the mouth and throat, respiratory ailments, skin disorders, tuberculosis, infections, etc. [9,10,11,12]. In addition, their leaves and EOs have been widely applied for culinary purposes since ancient times (e.g., as spices and flavor additives), in perfumery, and in cosmetics [9]. Over the years, many *Salvia* taxa (species and subspecies) have been studied for their chemical diversity, unveiling a rich reservoir of diversified and specialized products, including volatile compounds (essential oil; monoterpenoids, sesquiterpenes, etc.) and nonvolatile compounds (e.g., terpenoids, flavonoids, and phenolic acids) [12,13]. *Salvia* EOs have attracted increasing interest for their potential beneficial effects on human health, demonstrating to date various pharmacological activities, including strong antimicrobial and anti-inflammatory potential among others [12,14].

According to different pharmacopeias (United States, British, and European), the quality control of EOs from medicinally important plants is essential and involves the evaluation of any qualitative and quantitative modification of their constituents, particularly the principal and active ones [15]. It is noteworthy to mention that high variability in the chemical composition of *Salvia* EOs has been observed, which is usually attributed to various factors such as genotypic variation or different geographical origin, varied environmental conditions, diversified harvesting time, and different types of plant material (e.g., fresh or dried/cultivated or collected from wild populations, different plant parts, etc.), thus resulting in the high chemical polymorphism of the EOs of the studied materials and subjects (e.g., [16,17,18]).

Context-wise, the scope of this study was three-fold; firstly, we aimed to detect variability patterns in EO composition between wild-growing and cultivated pairs of samples from members in four *Salvia* sections or three clades such as *S. argentea* L. (Sect. *Aethiopis*; Clade I-C), *S. ringens* Sm. (Sect. *Eusphace*; Clade I-D), *S. verticillata* L. (Sect. *Hemisphace*; Clade I-B), *S. amplexicaulis* Lam., and *S. pratensis* L. (Sect. *Plethiosphace*; Clade I-C). Secondly, we examined various samples from wild-growing populations of different species to document natural variability in EO composition due to different geographical and environmental conditions by employing members of three *Salvia* sections or two clades, namely *S. sclarea* L. (six samples; Sect. *Aethiopis* or Clade I-C), *S. ringens* (three samples; Sect. *Eusphace* or Clade I-D) and *S. amplexicaulis* (five samples; Sect. *Plethiosphace* or Clade I-C). Furthermore, we examined the EO composition of four wild-growing species of two *Salvia* sections, i.e., *S. aethiopis* L., *S. candidissima* Vahl, and *S. teddii* of Sect. *Aethiopis*, as well as the cultivated material of *S. virgata* Jacq. of Sect. *Plethiosphace*, all belonging to Clade I-C. All results of these investigations were chemometrically evaluated to identify the sample relationships based on their chemical classes. Taken together, the findings of previous studies are summarized and critically discussed with the obtained results.

## 2. Results and Discussion

### 2.1. Chemical Analysis of EOs

A total number of 26 *Salvia* population samples (20 wild-growing and 6 cultivated) were studied, out of which 24 were collected from Greece and 2 from North Macedonia (Supplementary Materials Table S1). The plants were collected at different wild habitats at diverse altitudes spanning from 82 to 1532 m (Supplementary Materials Table S1). The EO yields of the studied materials (Supplementary Materials Table S1) ranged from 0.50 to 1.50% (*v*/*w*, based on the dry weight of the plant material), with the sample of *S. candidissima* presenting the highest amount (1.50%). In total, 205 compounds were identified in the present analysis (Table 1, Table 2, Table 3 and Table 4).

A summary of the main chemical compounds (>5.0%) and groups of the investigated *Salvia* EOs reported in the literature is presented in Supplementary Materials Table S2. In total, 74 publications were retrieved from 1976 to 2023.

#### 2.1.1. *Salvia* Members of Section *Aethiopis*/I-C Clade

Overall, 34 compounds were identified in the *S. aethiopis* EO (saeth), representing 98.1% of the total components (Table 1). The main chemical constituents (>5.0%) were (E)-caryophyllene (30.6%), germacrene D (20.0%), α-copaene (13.7%), caryophyllene oxide (8.4%), and α-humulene (6.4%). The EO presented high amounts of sesquiterpene hydrocarbons (82.2%), followed by oxygenated sesquiterpenes (13.4%) (Table 1). Oxygenated diterpenes were found in low amounts (1.3%).

Previous studies have investigated the EO of *S. aethiopis* from Iran [18,19,20,21], Serbia [22,23,24], Spain [25], Turkey [26,27,28], and former Yugoslavia [29,30] (Supplementary Materials Table S2). Our results were similar to the previous findings indicating (E)-caryophyllene (=β-caryophyllene) as the major component, but with some differences in the percentages of the rest of the main compounds [19,20,21,30]. It is noteworthy to mention that bicyclogermacrene and bornyl acetate were found in low amounts (0.6% and 0.3%, respectively), while linalool was not detected in our study. Sesquiterpenoids were also the main chemical classes, followed by monoterpenoids in previous works (Supplementary Materials Table S2). This study represents the first report on *S. aethiopis* EO collected from Greece.

The chemical constituents of the EOs of two *S. argentea* population samples, one cultivated (sargc1) and one wild-growing (sarg2), are presented in Table 1. A total percentage of 95.9% (sargc1) and 99.1% (sarg2), represented by 59 and 76 compounds, respectively, were identified in the EOs. In both samples, sesquiterpene hydrocarbons were the major chemical class, with 30.8% and 46.4%, respectively (Table 1). However, quantitative variations were observed in the rest of the main categories. For instance, the second main group of the cultivated sample (sargc1) was alkanes (29.9%), while in the wild-growing population (sarg2), it was oxygenated monoterpenes (20.3%). Further differences were noticed in the principal constituents (>5.0%) among the two samples. In the cultivated sample (sargc1) from propagated wild-growing material, tetracosane (20.0%), germacrene D (13.6%), pentacosane (8.8%), and bornyl acetate (6.8%) were the predominant compounds. By contrast, germacrene D (20.0%), bornyl acetate (10.4%), δ-cadinene (5.9%), α-pinene (5.7%), and α-copaene (5.4%) were identified as the main components in the wild-growing population (sarg2).

Previous studies have investigated the EO of *S. argentea* from Greece [31], Italy [32], Morocco [32,33], Serbia [34], North Macedonia [35], Tunisia [36,37], and Turkey [38] (Supplementary Materials Table S2). It is worth noting that many differences were observed across the results of these studies. More specifically, oxygenated sesquiterpenes were the predominant chemical class in most of the Mediterranean samples from Italy [32], North Macedonia [32,35], Serbia [32,34], and Tunisia [36], while the samples from Morocco [32,33] and Turkey [38] were characterized by oxygenated monoterpenes (64.1%) and monoterpene hydrocarbons (24.26%), respectively. Regarding the main components of EOs, caryophyllene oxide (37.6%) was identified as the main compound in the samples from North Macedonia, followed by α-copaene (8.5%), humulene epoxide II (6.3%), and *β*-caryophyllene (6.1%) [32,35], whereas in the sample from Serbia, viridiflorol (32.4%), manool (14.6%), α-humulene (10.7%), and *cis*-thujone (7.3%) were identified [34]. The EOs of the two Tunisian populations [36] were quite similar to the Serbian one. Although the composition of the EO of *S. argentea* collected in Sicily was rich in oxygenated sesquiterpenes like those from North Macedonia, Serbia, and Tunisia, quite different constituents were found. More precisely, 14-hydroxy-*α*-humulene (40.1%), 1,3,8-*p*-menthatriene (12.1%), globulol (7.4%), and *β*-sesquiphellandrene (5.8%) were the main components of the Sicilian sample, which were not present in the other EOs. The effect of different growth stages on the composition of the *S. argentea* EO has been also studied in samples collected from Tunisia, reporting observed differences in the chemical classes and constituents among the three developmental stages [37]. In the EO from Turkey, sclareol (40.01%), germacrene D (13.90%), *β*-pinene (11.93%), sclareol oxide (9.65%), and *α*-pinene (6.59%) were the main compounds [38]. Moreover, germacrene D (37.41%) and *β*-caryophyllene (6.75%) were reported as major constituents in the EO from Greece [31]. Although germacrene D was also the principal component in our samples, variations were found in the composition of the total EOs. Overall, our findings presented differences from the results of previous studies regarding the main chemical classes and constituents of EOs, which could be attributed to environmental conditions, harvesting time, the type/origin of the plant material and plant parts examined. It is noteworthy to mention that this study is the first report on *S. argentea* EOs collected from Greece, investigating two different population samples (cultivated and wild-growing).

In total, 75 components were identified in *S. candidissima* EO, constituting 84.4% of the total oil (Table 1). The most abundant constituents (>5.0%) were germacrene D (15.9%), β-pinene (9.7%), α-pinene (7.5%), sabinene (6.2%), spathulenol (5.3%), and sclareol (5.1%). Monoterpene hydrocarbons (28.0%) were the major components in the EO, followed by sesquiterpene hydrocarbons (27.1%), oxygenated sesquiterpenes (14.9%), oxygenated diterpenes (7.6%), and oxygenated monoterpenes (6.1%).

Previously, Pitarokili et al. [39] investigated the *S. candidissima* EO from Greece (Supplementary Materials Table S2). Although monoterpenes were also the predominant class, the main compounds were *α*-pinene (11.2%), 1,8-cineole (9.9%), *p*-cymene (7.4%), myrtenal (6.5%), pinocarvone (6.2%), camphene (5.7%), and *trans*-pinocarveol (5.5%). Interestingly, germacrene D was the dominating compound in the sample studied here, whereas it was not found in the previous study. As a result, variations were evidenced in the amount of the EO constituents compared to our results. This could probably be attributed to the different geographical areas sampled and the year of the sample collection. Moreover, the previously investigated EO of *S. candidissima* collected from Turkey [40,41,42] (Supplementary Materials Table S2) revealed camphor (28.94%), bornyl acetate (12.80%), borneol (9.44%), β-cadinene (5.88%), *α*-caryophyllene (5.40%), and 1,8-cineole (5.15%) as the major constituents, with oxygenated monoterpenes being the principal group in one study [41]. However, spathulenol (12.75%), caryophyllene oxide (8.67%), ledene oxide (6.98%), and *o*-cymene (6.03%) were only detected in another previous study [42].

Overall, 37 volatile constituents were identified in the EO of *S. teddii*, representing 94.4% of the total oil (Table 1). The EO was characterized by a high content of (E)-caryophyllene (59.6%), followed by caryophyllene oxide (11.2%), germacrene D (6.2%), and (E)-*β*-farnesene (5.4%). The sesquiterpene fraction was the main group of the compounds (93.9%), of which sesquiterpene hydrocarbons were the prevailing group (80.2%) compared to the oxygenated sesquiterpenes (13.7%). This study reports the composition of *S. teddii* EO for the first time; thus, no data are available for any comparison.

Overall, 98 compounds were found, representing a range of 92.1–98.7% of the EOs in the six samples of *S. sclarea* (sscl1–sscl6) collected from different areas of Greece (Supplementary Materials Table S1). Their chemical constituents are listed in Table 1. The qualitative oil compositions did not present any differences; however, quantitative variation was observed. The most abundant constituents were linalool acetate (21.2–25.7%), linalool (13.6–24.5%), germacrene D (5.1–14.7%), sclareol (7.6–11.2%), and *α*-terpineol (4.8–7.4%). All the EOs were characterized by a high content of oxygenated monoterpenes (47.1–62.8%), followed by sesquiterpene hydrocarbons (9.6–26.1%), oxygenated diterpenes (10.6–14.2%), and oxygenated sesquiterpenes (5.0–7.4%).

Previous studies have been carried out in the *S. sclarea* EO from Egypt [43], France [44], Germany [45], Greece [46,47,48,49], Iran [18,50,51], Italy [52,53,54,55], Lebanon [56], Poland [57], Serbia [24,58], Slovakia [59], Spain [25], Uruguay [15], Tajikistan [60], Turkey [28,61,62], and Uzbekistan [63,64] (Supplementary Materials Table S2). Linalool and linalyl acetate were reported as the most characteristic and dominant volatiles of clary sage oil, ranging between 6.5–24.0% and 56.0–78.0%, respectively, while the amount of sclareol varied from 0.4% to 2.6% [48]. It is noteworthy to mention the presence of sclareol in considerable amounts in all the Greek samples herein examined (7.6–11.2%). Our results were generally in accordance with previous reports on *S. sclarea* EO from Greece [46,47,48,49]. However, there are noticeable variations in terms of the relative percentages of the components. According to Sharopov and Setzer [60], different chemotypes have been found in *Salvia* EOs such as linalyl acetate/linalool, geraniol/geranyl acetate, methyl chavicol, and germacrene D chemotypes. The profiling of the samples studied here could be categorized as the linalyl acetate/linalool chemotype.

#### 2.1.2. *Salvia* Members of Section *Eusphace*/I-D Clade

In total, 112 components were identified in all four *S. ringens* EOs examined, representing 99.6% (src1), 98.1% (sr2), 99.4% (sr3), and 97.5% (sr4) of the oils. The chemical constituents identified in the EOs are listed in Table 2. The EOs were similar regarding the qualitative pattern but displayed some quantitative differences. The major constituents were 1,8-cineole (17.9–40.2%), *α*-pinene (9.1–12.5%), camphene (5.5–15.6%), *β*-pinene (4.8–7.7%), bornyl acetate (2.0–20.3%), and borneol (1.6–12.9%). Intriguingly, bornyl acetate was the principal compound only in the src1 sample, which was derived from the cultivated material originating from wild-growing populations. However, 1,8-cineole was the major constituent in the rest of the wild-growing samples (sr2-sr4). All the EOs were characterized by a high content of oxygenated monoterpenes (48.2–58.1%), followed by monoterpene hydrocarbons (31.4–36.7%) (Table 2).

The results of this study are generally in agreement with those of previous studies examining the EO of *S. ringens* from Greece [65,66] (Supplementary Materials Table S2). However, there are important variations in terms of the relative percentages of the components. More specifically, 1,8-cineole, *α*-pinene, bornyl acetate, and *β*-pinene were the main compounds of the EO in the first study [65]; it is noteworthy to mention that bornyl acetate was found in fewer amounts in all our analyzed samples, apart from src1. In the second study, *α*-pinene, *β*-pinene, 1,8-cineole, camphene, and borneol were detected as the dominant components [66]. Moreover, the major component of *S. ringens* EO from Bulgaria was camphor [67] (Supplementary Materials Table S2). In our findings, camphor was only identified in low amounts (1.8–4.5%). In the EO of North Macedonia, 1,8-cineole, camphene, borneol, and *α*-pinene were the main compounds [68] (Supplementary Materials Table S2). However, our results of src1, which was ex situ-cultivated in Northern Greece after the propagation of cuttings from wild-growing populations in North Macedonia, revealed different dominant components such as bornyl acetate (20.3%), 1,8-cineole (17.9%), camphene (15.6%), borneol (12.9%), *α*-pinene (9.1%%), and *β*-pinene (4.8%). It is well established that such differences in the chemical composition might be the result of several factors, including the plant part or developmental stage examined, origin, and the harvesting period [68].

#### 2.1.3. *Salvia* Members of Section *Hemisphace*/I-B Clade

The chemical constituents of the EOs of two *S. verticillata* populations, one cultivated (sverc1) but originally sourced from the wild and one wild-growing (sver2), are presented in Table 3. Total percentages of 92.4% (sverc1) and 97.4% (sver2) represented by 45 and 59 compounds, respectively, were identified in the EOs. The two EO compositions presented qualitative and quantitative differences. In sverc1, (E)-nerolidol (35.0%), germacrene D (11.5%), and *β*-pinene (6.1%) were the main constituents, whereas 4aα,7α,7aα-nepetalactone (51.4%), 1,8-cineole (18.4%), and caryophyllene oxide (6.0%) were the dominating components in sver2. Oxygenated sesquiterpenes (50.5%) were the major group, followed by sesquiterpene hydrocarbons (30.1%) and monoterpene hydrocarbons (9.6%) in sverc1. In contrast, sver2 was characterized by a high content of oxygenated monoterpenes (74.2%).

According to our literature survey on previous studies on *S. verticillata* EOs (Supplementary Materials Table S2), their composition is reported to be characterized by a high level of complexity. More specifically, a previous study [39] investigated the EO of *S. verticillata* from Greece and reported monoterpenes as the major fraction of the oil with the main constituents of *β*-pinene (30.7%), *p*-cymene (23.0%), isopropyl ester of lauric acid (16.8%), *α*-pinene (7.6%), and (E)-nerolidol (5.2%). It is noteworthy to mention that the isopropyl ester of lauric acid was not found in our samples. The *S. verticillata* EO from Iran has been extensively studied [18,69,70,71,72,73,74]. These EOs mainly consisted of sesquiterpenes, such as *β*-caryophyllene, germacrene D, bicyclogermacrene, and *α*-humulene [74,75]. In addition, *β*-caryophyllene (13.3%) and *γ*-muurolene (10.3%) were the principal compounds in the EO from former Yugoslavia [30]. *S. verticillata* EO from three different populations in Serbia exhibited some differences among the samples [24,76]. Moreover, germacrene D, bicyclogermacrene, and β-caryophyllene were the dominant constituents of the EO of this species from Italy [77]. In the EO from Poland, *α*-pinene (10.72%), camphor (5.23%), and limonene (5.85%) were the main compounds [57]. Smekalova et al. [78] investigated the chemical compositions of *S. verticillata* EO collected from seven locations in the Czech Republic. Consequently, a high chemical polymorphism could be noticed in the *S. verticillata* EO due to the geographical origin of the studied materials, the processed plant parts examined, and the applied techniques [75,77]. Further studies are necessary to better understand the variability in the chemical profile of *S. verticillata*.

#### 2.1.4. *Salvia* Members of Section *Plethiosphace*/I-C Clade

In total, 87 components were identified in the six *S. amplexicaulis* EOs studied, representing 90.7% (samp1), 89.9% (samp2), 87.8% (samp3), 94.6% (samp4), 99.6% (samp5), and 93.6% (sampc6) of the oils. The chemical constituents identified in the EOs are presented in Table 4. The EOs were similar regarding the qualitative pattern but displayed quantitative differences. In the wild-growing samples (samp1-samp5), the major constituents were germacrene D (4.0–40.2%), caryophyllene oxide (6.8–35.1%), and (E)-caryophyllene (5.7–14.8%). It is worth mentioning that samp1 showed remarkable variations in the main compounds compared to the others since caryophyllene oxide (35.1%) was the predominant compound, followed by salvial-4(14)-en-1-one (7.0%), vulgarol B (6.4%), and E-caryophyllene (5.7%). Furthermore, spathulenol was found in all the samples; however, it was identified as the major constituent, with 18.7% only in samp2. Viridiflorol was detected in a high amount (12.1%) in samp4 but in low percentages in samp2 (0.6%) and samp3 (0.4%). The ex situ-cultivated sample (sampc6) originating from wild-growing individuals demonstrated a different EO profile, with 1,8-cineole (19.6%), *α*-pinene (14.4%), camphene (12.9%), and *β*-pinene (8.6%) being the main compounds. Intriguingly, camphene and *β*-pinene were found only in this sample, while germacrene D was in traces. Sesquiterpenes were the major group in the EOs from the wild-growing samples, with oxygenated sesquiterpenes being in higher amounts in samp1 (64.5%), samp2 (61.8%), and samp4 (45.4%), whereas sesquiterpene hydrocarbons were predominant in samp3 (58.4%) and samp5 (72.2%) (Table 4). Monoterpenes characterized the EO of the ex situ-cultivated sample (sampc6) of plant material originating from the wild, with monoterpene hydrocarbons being at 39.6% and oxygenated monoterpenes being at 21.1%. Oxygenated diterpenes were detected in all samples, ranging from 0.1% to 5.5%.

Considering the *S. amplexicaulis* EO from Poland [57] and Serbia [24,79,80] (Supplementary Materials Table S2), sesquiterpenes were found to be the dominant chemical class in the EOs reported in two of the previous studies [79,80], with detected monoterpene hydrocarbons as the main group in another one [24]. Regarding the major constituents, variations were observed among the reported studies, which could be attributed to the different collection areas of the plant materials and the applied techniques. This study reports the composition of *S. amplexicaulis* EO from different parts of Greece for the first time.

The chemical constituents of the two EOs of *S. pratensis* subsp. *pratensis* are presented in Table 4. However, it should be mentioned that one of these samples refers to ex situ-cultivated individuals originally collected from wild-growing populations in North Macedonia (sprc1), which were identified as *S. pratensis* subsp. *bertolonii* (Vis) Soó but are included in Table 4 and Supplementary Materials Tables S1 and S2 as *S. pratensis* subsp. *pratensis* to adopt the nomenclature of the Plants Of the World Online [81], while another one (spr2) refers to the typical wild-growing population identified as *S. pratensis* subsp. *pratensis*. Similar total percentages of 97.4% (sprc1) and 97.9% (spr2) represented by 25 and 19 compounds, respectively, were found in the EOs of these samples. In both samples, sesquiterpene hydrocarbons were the major chemical class, with percentages of 48.8% and 53.6%, respectively (Table 4), thus showing a similar profile in terms of dominant compounds. However, quantitative variations were observed regarding the rest of the main chemical classes. For instance, the second main group of sprc1 was oxygenated sesquiterpenes (29.0%), while in spr2, it was monoterpene hydrocarbons (27.2%). In contrast, similarities were also detectable; in both EOs (sprc1 and spr2), the major compounds were (E)-caryophyllene (45.5%; 46.9%), caryophyllene oxide (25.8%; 10.4%), and sabinene (14.8%; 15.0%).

Anackov et al. [82] have investigated the EOs of *S. pratensis* subsp. *pratensis* from Serbia (Supplementary Materials Table S2). Caryophyllene oxide and (Z)-caryophyllene were the dominant constituents in one sample attributed to the EO of *S. bertolonii* Vis., which is considered a synonym of *S. pratensis* subsp. *pratensis*, with oxygenated sesquiterpenes being the most abundant class. However, the EO from the typical *S. pratensis* subsp. *pratensis* from a different Serbian region had (E)-caryophyllene (26.4%), (Z)-*β*-farnesene (6.0%), *β*-cubebene (5.6%), and epi-bicyclosesquiphellandrene (5.6%) as the major components in the same study, with sesquiterpene hydrocarbons being in higher amounts. In addition, camphene and thujol were found as the main constituents in the *S. pratensis* EO from Poland [57]. Such differences and similarities clearly indicate the need for new multidisciplinary (taxonomic, genetic, chemical, and ecological) investigations that should be carried out in an attempt to shed light on the circumscription of *S. pratensis* subsp. *bertolonii* at the subspecies or species level.

In total, 39 compounds were identified in the EO of ex situ-cultivated *S. virgata* from Greece originating from wild-growing populations, which presented about 99.6% of the total composition of the oil (Table 4). The major constituents were sabinene (21.2%), (E)-caryophyllene (20.8%), allo-aromadendrene (15.2%), caryophyllene oxide (6.6%), and germacrene D (6.1%). Sesquiterpene hydrocarbons (46.3%) were the dominant chemical class, followed by monoterpene hydrocarbons (34.1%), oxygenated sesquiterpenes (12.7%), and oxygenated monoterpenes (5.4%) (Table 4).

Previous studies were performed on the EO of *S. virgata* from Iran [18,83,84,85,86,87,88,89,90] and Turkey [28,91,92] (Supplementary Materials Table S2). Overall, E-caryophyllene and caryophyllene oxide have mainly been found as the major components, while sesquiterpenes were the principal group in the *S. virgata* EOs from Iran [74]. Interestingly, some studies [86] investigated EOs from leaves, stems, and aerial parts separately and reported that two oils (leaf EO and EO from flowering aerial parts) were mainly characterized by sesquiterpene hydrocarbons, while in the stem oil, fatty acids predominated over monoterpenes and sesquiterpenes. Another study [87] showed that the different harvest times (pre-flowering and full-flowering stages) affected oil yield, and the highest oil yield was observed in the flowering stage, with the main components of the oil being *β*-caryophyllene (24.58–42.54%), caryophyllene oxide (10.25–19.88%), sabinene (8.64–19.58%), 1-octen-3-ol (7.54–8.59%), terpinene-4-ol (4.25–6.64%), and *α*-thujene (3.74–6.46%). However, samples from Turkey presented different EO profiles (Supplementary Materials Table S2). For instance, 1,8-cineole (20.3%), *α*-copaene (18.6%) and germacrene D (17.6%) were determined as the major compounds of *S. virgata* EO in one study [92], whereas borneol (23.41%), palmitic acid (7.93%) and *trans*-pinocarvyl acetate (5.06%) were the main constituents in another [28]. Hence, important variability in terms of EO compositions in this species was evidenced based on geographical origin and examined plant parts. Although the overall qualitative EO profile of the sample herein studied was similar to that reported in the literature, quantitative differences were detected. This study reports the composition of *S. virgata* EO from Greece for the first time.

### 2.2. Chemometric Analysis

**Figure 1:**
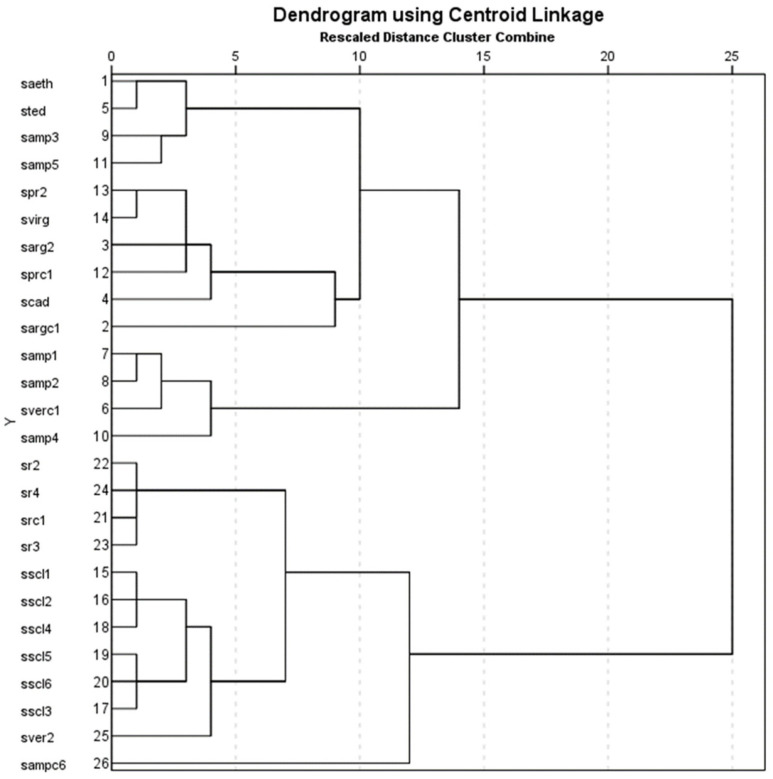
Hierarchical cluster analysis of 26 samples of *Salvia* members of three phylogenetic clades or four taxonomic sections. For the coding of samples, see Table 1, Table 2, Table 3, and Table 4. For the arrangement of *Salvia* species in taxonomic sections and phylogenetic clades, see Table 5.

The hierarchical cluster analysis (HCA) (Figure 1) showed the formation of two major groups for the studied *Salvia* samples. The first group (Group I) comprised 14 samples clustering the studied members of Sect. *Aethiopis* (saeth, sargc1, sarg2, scad, and sted) together with members of Sect. *Plethiosphace* (samp1, samp2, samp3, samp4, samp5, sprc1, spr2, and svirg), all belonging to the phylogenetic Clade I-C; however, in the same major group, the cultivated sample of *S. verticillata* (sverc1) of Sect. *Hemisphace* was also clustered. The second group (Group II) comprised 12 samples clustering *S. sclarea* samples of Sect. *Aethiopis* or Clade I-C in a subgroup (sscl1, sscl2, sscl3, sscl4, sscl5, and sscl6), with *S. ringens* samples of Sect. *Eusphace* or clade I-D (src1, sr2, sr3, and sr4) in another subgroup; however, the wild-growing sample of *S. verticillata* of Sect. *Hemisphace* or Clade I-B (sver2) and the sample of cultivated *S. amplexicaulis* of Sect. *Plethiosphace* or Clade I-C (sampc6) were not tightly clustered with the rest.

**Figure 2:**
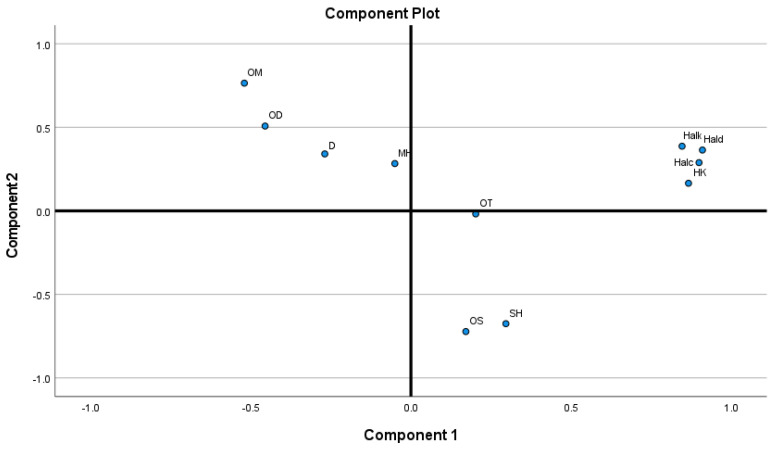
Contributions of chemical classes to principal components.

The PCA elucidated 82% of data variability. The main contribution to the first principal component (PC) was observed for HAld, HAlc, HK, and Halk. Positive loadings were observed for SH, OS, HAlc, HAld, HK, HAlk, and OT, while negative loadings were found for MH, OM, and D. The main contribution to the second PC was OM and OS. Positive loadings were observed for MH, OM, D, OD, HAlc, HAld, HK, and HAlk, while negative for SH, OS, and OT (Figure 2). Additionally, the main contribution to the third PC was MH and OT. Positive loadings were observed for MH, OM, OS, HK, and OT, while negative loadings were found for SH, D, OD, HAlc, HAld, and HAlk. Finally, the main contribution of the fourth PC was OT and OD. Positive loadings were observed for OS, D, OD, HK, and OT, while negative loadings were observed for MH, OM, SH, HAlc, HAld, and HAlk. More details on the correlations can be found in the Supplementary Materials Figure S1.

Group I (saeth, sargc1, sarg2, scad, sted, samp1, samp2, samp3, samp4, samp5, sprc1, spr2, svirg, and sverc1) was characterized by the highest amounts of SH (19.1–82.2%), followed by OS (4.8–64.5%), OD (10.6–14.2%); smaller amounts of MH (0.2–34.1%), OM (0.1–20.3%), and HAlk (0–29.9%); and minor amounts of OD (0–7.6%), HAlc (0–3.4%), HAld (0–3.4%), HK (0–2.5%), OT (0–1%), and D (0–0.4%). Group II (sscl1, sscl2, sscl3, sscl4, sscl5, sscl6, src1, sr2, sr3, sr4, sver2, and sampc6) was characterized by the highest amounts of OM (21.1–74.2%), followed by MH (2.8–39.6%), SH (2.9–26.1%); smaller amounts of OS (1.9–16.6%) and OD (0–14.2%); and minor amounts of D (0–1.5%), OT (0–1.9%), HK (0–1.3%), HAlc (0–0.2%), HAld (0–0.05%), and HAlk (0–0.05%).

**Figure 3:**
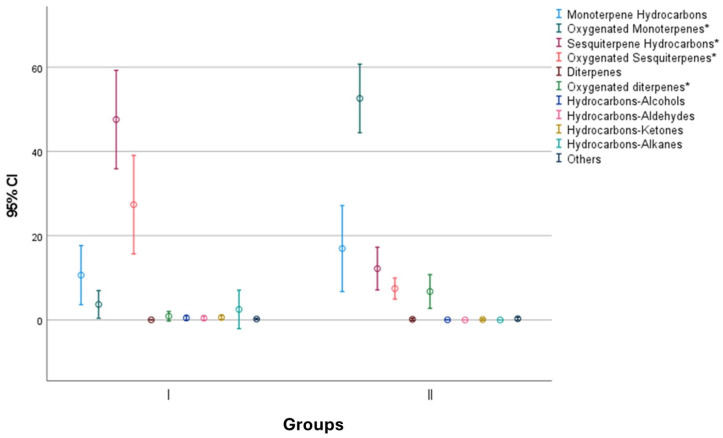
Chemical classes of two groups concerning the 26 samples of the 10 studied members of genus *Salvia* from different taxonomic sections or phylogenetic clades (Table 5 and Supplementary Materials Table S1). Mean and 95% confidence intervals are given. Chemical classes marked with asterisks (*) differed statistically significantly in the *t*-test (*p* < 0.05).

The analysis of the mean contents and standard deviations of the chemical classes showed that Group I was statistically different (*t*-test, *p* < 0.05) from Group II by the content of OM (I = 3.7 ± 5.7%; II = 52.6 ± 12.8%), SH (I = 47.6 ± 20.3%; II = 12.2 ± 8%), OS (I = 27.4 ± 20.2%; II = 7.5 ± 3.9%), and of OD (I = 0.9 ± 2%; II = 6.8 ± 6.3%) (Figure 3).

**Figure 4:**
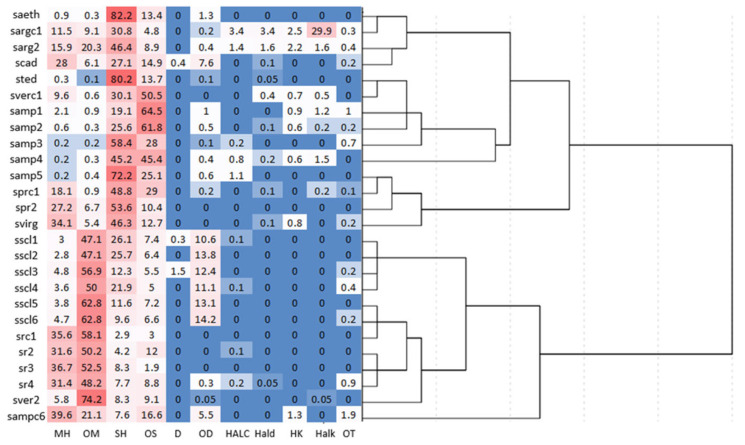
Clustered heat map of *Salvia* samples in chemical classes. For the coding of samples, see Table 1, Table 2, Table 3, and Table 4. For the arrangement of *Salvia* species in taxonomic sections and phylogenetic clades, see Table 5.

Applying additional multivariate analyses in the heatmap analysis combined with HCA with the chemical classes, the color pattern corresponding to different samples of *Salvia* members varied with color intensity and increased gradually, from lowest to the highest grade (blue indicates low correlations, while red color indicates high correlations). The clustered heatmap (Figure 4) confirmed the abovementioned clustering results for HCA and PCA.

## 3. Materials and Methods

### 3.1. Plant Materials

**Figure 5:**
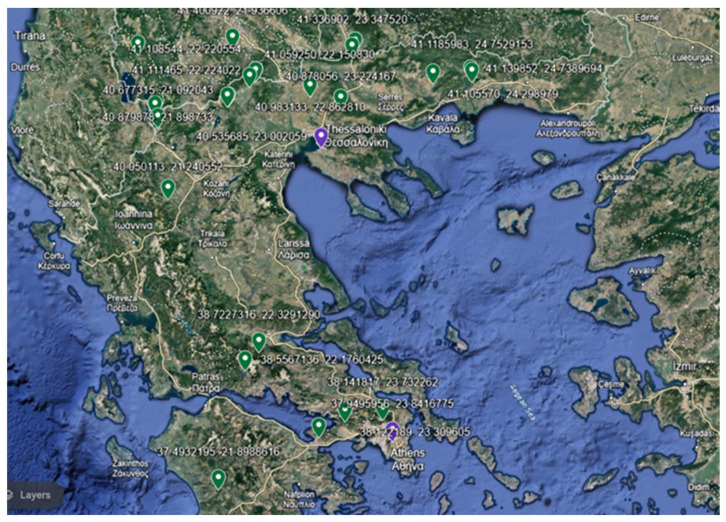
Botanical collections in Greece and the Republic of Northern Macedonia regarding the herein investigated *Salvia* species (*n* = 10) projected on Google Earth. Samples and living plant individuals collected from wild-growing populations are indicated with green symbols, while purple symbols indicate the location of ex situ cultivation sites for the plant individuals collected from the wild; these are close to Thessaloniki for *Salvia pratensis*, *S. amplexicaulis*, and *S. ringens* or close to Athens for *S. argentea*, *S. verticillata*, and *S. virgata* (see Supplementary Materials Table S1).

The flowering aerial parts of 10 *Salvia* species were collected from May to July 2021. Specifically, 24 samples originated from Greece and 2 from the Republic of North Macedonia (Figure 5). Among them, 20 samples were obtained from wild-growing populations (from 16 different areas of Greece), while 6 were sourced from ex situ-cultivated samples that originated from wild-growing populations (4 from Greece and 2 from North Macedonia). A detailed catalog of the samples, collection sites, and dates is provided in Supplementary Materials Table S1. Voucher specimens were identified by Dr. Nikos Krigas and are deposited in the Herbarium of the Balkan Botanic Garden of Kroussia (BBGK), Institute of Plant Breeding and Genetic Resources, Agricultural Organization Demeter, together with living plant specimens maintained ex situ (Supplementary Materials Table S1). The sections and clades of the investigated *Salvia* species are included in Table 5.

**Table 5:** Botanical names, sections, and clades of the 10 investigated *Salvia* species.

Taxon	Section ^1^	Clade ^2^
*Salvia aethiopis* L.	*Aethiopis*	I-C
*Salvia argentea* L.	*Aethiopis*	I-C
*Salvia candidissima* Vahl	*Aethiopis*	I-C
*Salvia sclarea* L.	*Aethiopis*	I-C
*Salvia teddii* Turill	*Aethiopis*	I-C
*Salvia ringens* Sm.	*Eusphace*	I-D
*Salvia verticillata* L.	*Hemisphace*	I-B
*Salvia amplexicaulis* Lam.	*Plethiosphace*	I-C
*Salvia pratensis* L.	*Plethiosphace*	I-C
*Salvia virgata* Jacq.	*Plethiosphace*	I-C

^1^ Section based on the study of Bentham [93]. ^2^ Section based on the study of Will & Glassen-Bockhoff [1].

### 3.2. EO Isolation

All collected plant materials were air-dried at room temperature for 10 days and then comminuted. About 20 g from each plant was used, and the EOs were obtained by hydro-distillation in a modified Clevenger apparatus for 3 h, according to the *Hellenic Pharmacopoeia* [94]. GC (gas chromatography) grade n-pentane was used for the collection of the EOs, with the addition of anhydrous sodium sulfate to reduce any moisture. The EOs were subsequently analyzed by GC–MS (gas chromatography–mass spectrometry) and finally stored at −20 °C.

### 3.3. GC–MS Analysis

GC–MS analyses were carried out using a Hewlett Packard 7820A-5977B MSD system operating in EI mode (70 eV), equipped with an HP-5MS fused silica capillary column (30 m × 0.25 mm; film thickness 0.25 µm), and a split–splitless injector. The temperature program was 60 °C at the time of the injection, and then it was raised to 300 °C at a rate of 3 °C/min and subsequently held at 300 °C for 10 min. Helium was used as a carrier gas at a flow rate of 2.0 mL/min. The injected volume of the samples was 1 μL. Each analysis was repeated three times.

Retention index (RI) values were calculated using a linear equation by Van den Dool and Kratz [95] based on a homologous series of n-alkanes from C9 to C24. The identification of the chemical components was based on a comparison of RI values and mass spectra fragmentation patterns with those reported in the NIST/NBS and Wiley libraries, as well as those described by Adams [96] and other literature data.

### 3.4. Chemometric Analysis

We applied hierarchical cluster analysis (HCA), using the centroid clustering method, with interval measuring the squared Euclidean distance and applying standardization to variables with range -1 to 1 to evaluate the variability of samples. We also applied principal components analysis (PCA) to investigate the amount of variability explained. The number of principal components included in the analysis was based on the percent of variability explained (criteria set to over 80%). The data consisted of 11 chemical classes: monoterpene hydrocarbons (MH), oxygenated monoterpenes (OM), sesquiterpene hydrocarbons (SH), oxygenated sesquiterpenes (OS), diterpenes (D), oxygenated diterpenes (OD), hydrocarbons–alcohols (HAlc), hydrocarbons–aldehydes (Hald), hydrocarbons–ketones (HK), hydrocarbons–alkanes (HAlk), and others (OT). Data on the total percentages of these chemical classes were evaluated. The total number of samples was *n* = 26. All analyses were performed using SPSS software (SPSS v.28 ΙΒΜ Corporation, 2023 Armonk, New York, USA).

### 3.5. Literature Review

An extensive survey concerning the EO and volatile compounds of each examined *Salvia* species was conducted separately in widely used scientific databases such as PubMed, Scopus, and Google Scholar using keywords related with EOs and volatile constituents (i.e., *Salvia aethiopis* essential oil, *Salvia aethiopis* chemical composition, *Salvia aethiopis* chemical profile, *Salvia aethiopis* volatile constituents, *Salvia amplexicaulis* essential oil, *Salvia amplexicaulis* chemical composition, *Salvia amplexicaulis* chemical profile, *Salvia amplexicaulis* volatile constituents, *Salvia argentea* essential oil, *Salvia argentea* chemical composition, *Salvia argentea* chemical profile, *Salvia argentea* volatile constituents, *Salvia candidissima* essential oil, *Salvia candidissima* chemical composition, *Salvia candidissima* chemical profile, *Salvia candidissima* volatile constituents, *Salvia pratensis* essential oil, *Salvia pratensis* chemical composition, *Salvia pratensis* chemical profile, *Salvia pratensis* volatile constituents, *Salvia ringens* essential oil, *Salvia ringens* chemical composition, *Salvia ringens* chemical profile, *Salvia ringens* volatile constituents, *Salvia sclarea* essential oil, *Salvia sclarea* chemical composition, *Salvia sclarea* chemical profile, *Salvia sclarea* volatile constituents, *Salvia teddii* essential oil, *Salvia teddii* chemical composition, *Salvia teddii* chemical profile, *Salvia teddii* volatile constituents, *Salvia verticillata* essential oil, *Salvia verticillata* chemical composition, *Salvia verticillata* chemical profile, *Salvia verticillata* volatile constituents, *Salvia virgata* essential oil, *Salvia virgata* chemical composition, *Salvia virgata* chemical profile, and *Salvia virgata* volatile constituents). In total, 74 publications were retrieved from 1976 to 2023, while articles focused on non-volatile constituents were excluded.

## 4. Conclusions

In this study, we provided new information regarding the quantitative and qualitative variation in EOs in wild-growing and cultivated pairs of samples from members in four different *Salvia* sections or three different clades, thus evidencing the profile of the cultivated samples compared to the ones sourced from the wild. On one hand, such differences and similarities may indicate the natural potential of the examined *Salvia* species in quantitative and qualitative terms, and this can pave the way for the sustainable exploitation of the cultivated materials; on the other hand, these findings clearly indicate the need for new multidisciplinary (taxonomic, genetic, chemical, and ecological) investigations employing *Salvia* members. This is especially true for any attempt to shed light on synonymizing *S. pratensis* subsp. *bertolonii* with subsp. *pratensis* or, in contrast, to provide solid evidence regarding the re-circumscription of *S. pratensis* at the subspecies or species level. Furthermore, in this investigation, we provided documentation regarding the natural variability in EO composition due to different genotypes adapted in different geographical and environmental conditions by employing members of three different *Salvia* sections or two different phylogenetic clades.

By reviewing published research and through a comprehensive re-assessment of the EO composition of the *Salvia* species herein studied in terms of taxonomic sections and phylogenetic clades, important variability patterns were discussed, providing evidence regarding the effects of genotypes adapted in different geographical origins and environmental conditions, revealing diversification patterns of EO composition between cultivated and wild-growing samples, and documenting EO variability in the different plant parts examined.

Novelty-wise, this investigation outlined two distinct groups (Group I and Group II) based on the chemical classes among 10 *Salvia* members of different taxonomic sections or phylogenetic clades, which were quantitatively and qualitatively discerned; these can be further explored with the help of classical or modern taxonomic studies on *Salvia*. Moreover, this study furnished new data regarding the chemotaxonomy of *Salvia* members of Sect. *Aethiopis* and Sect. *Plethiosphace* of Clade I-C; it represents the first report from Greece on the EO composition of *S. aethiopis* (Sect. *Aethiopis*) and *S. argentea* originated from two different population samples (cultivated and wild-growing, Sect. *Aethiopis*), as well as the first Greek report for *S. amplexicaulis* (from different Greek regions) and *S. virgata* (Sect. *Plethiosphace*), all phylogenetically belonging to Clade I-C. Rich sclareol content is consolidated for Greek *S. sclarea.* Additionally, this study exclusively reports the EO composition of *S. teddii* for the first time (Sect. *Aethiopis*; Clade I-C).

Regarding the benefits of the investigated *Salvia* EOs on human health, the relevant studies reviewed in this paper for the specific taxa were found to be limited. Indeed, EOs of some species have not been pharmacologically studied so far. In contrast, *S. sclarea* EO has been explored for its antimicrobial activity in vitro. However, additional experimental studies should be carried out in order to determine the possible applications of these EOs in human health.

## Figures and Tables

**Table 1:** Chemical composition of the studied *Salvia* essential oils belonging to Sect. *Aethiopis*/I-C clade (saeth: *S. aethiopis*, sarg: *S. argentea*, sted: *S. teddii*, sscl: *S. sclarea*).

No	RI_C_	RI_L_	Compounds	saeth	sargc1	sarg2	scad	sted	sscl1	sscl2	sscl3	sscl4	sscl5	sscl6
				Percentage (%)										
1	920	921	Tricyclene		0.1	0.2	tr							
2	924	924	*α*-Thujene		tr	tr	0.2	tr						
3	930	932	*α*-Pinene	0.3	4.3	5.7	7.5	tr			0.1	tr		tr
4	944	946	Camphene	0.1	2.0	3.0	0.8				tr			tr
5	968	969	Sabinene	0.3	0.7	0.9	6.2	0.2				tr		tr
6	972	974	*β*-Pinene	0.2	3.2	4.3	9.7	0.1			0.1			0.1
7	975	977	1-Octen-3-ol						0.1			0.1		
8	988	988	Myrcene		0.2	0.3	0.2		0.9	0.8	1.4	1.2	1.2	1.4
9	1001	1002	*α*-Phellandrene				0.1							
10	1013	1014	*α*-Terpinene		0.1	0.2	0.2				tr	tr		tr
11	1023	1024	Limonene		0.5	0.6	2.0		0.3	0.3	0.4	0.4	0.4	0.4
12	1025	1026	1,8-Cineole	tr				0.1						
13	1032	1032	(Z)-*β*-Ocimene						0.5	0.5	0.8	0.7	0.6	0.8
14	1043	1044	(E)-*β*-Ocimene				tr		1.0	0.9	1.5	1.3	1.2	1.5
15	1052	1054	*γ*-Terpinene		0.2	0.3	0.6				tr	tr		tr
16	1063	1065	*cis*-Sabinene hydrate				0.2							
17	1083	1086	Terpinolene		0.1	0.2	0.2		0.3	0.3	0.5	tr	0.4	0.5
18	1087	1089	*p*-Cymenene		0.1	0.2	0.3	tr						tr
19	1095	1095	Linalool		0.5	3.5	0.7	tr	14.6	13.6	18.2	16.3	24.5	21.7
20	1096	1098	*trans*-Sabinene hydrate				0.2							
21	1098	1100	*n*-Nonanal		3.2	1.5	0.1	tr						
22	1115	1118	*cis*-*p*-Menth-2-en-1-ol				0.1					tr		
23	1119	1122	*α*-Campholenal		0.1	0.4	0.3							
24	1126	1128	allo-Ocimene								tr	tr		tr
25	1131	1135	*trans*-Pinocarveol		0.1	0.5	0.4							
26	1135	1137	*trans*-Sabinol				0.4							
27	1138	1140	*trans*-Verbenol		0.1	0.4	0.3							
28	1139	1141	Camphor		tr	0.3								tr
29	1149	1154	Nerol oxide								tr			tr
30	1156	1160	Pinocarvone		tr	0.4	0.4							
31	1161	1165	Borneol		0.6	2.5	0.8				0.1			tr
32	1167	1170	*cis*-Linalool oxide			0.1					0.1			
33	1171	1174	Terpinen-4-ol		0.2	0.4	0.7		0.1		0.1	tr		0.1
34	1184	1186	*α*-Terpineol		0.1	0.1	0.3		5.1	4.8	6.2	6.1	7.4	6.8
35	1189	1194	Myrtenol		0.2	0.2	0.4							
36	1192	1195	Myrtenal			0.4	0.4							
37	1198	1200	*trans*-Dihydrocarvone											tr
38	1200	1201	*n*-Decanal		0.2	0.1								
39	1202	1204	Verbenone				0.1							
40	1205	1207	*trans*-Piperitol				tr							
41	1210	1214	Linalool formate						0.1			0.1		0.1
42	1211	1215	*trans*-Carveol				0.1							
43	1223	1227	Nerol						0.8	0.6	1.1	1.1	0.9	1.2
44	1230	1235	Isobornyl formate			0.1								
45	1234	1239	Carvone				tr							
46	1250	1254	Linalool acetate		0.2	0.2			21.2	23.9	24.4	22.3	24.1	25.7
47	1277	1280	Neryl formate									tr		
48	1279	1284	Bornyl acetate	0.3	6.8	10.4	0.2				tr			
49	1289	1289	Thymol								0.2			0.1
50	1295	1298	Carvacrol		tr	0.2								
51	1298	1298	Geranyl formate						0.1			0.1		0.1
52	1330	1335	*δ*-Elemene					0.9				0.2		0.2
53	1342	1348	*α*-Cubebene	0.4				tr	0.2					
54	1350	1356	Eugenol									tr		
55	1355	1357	4aα,7α,7aα-Nepetalactone								0.1			tr
56	1359	1359	Neryl acetate						1.6	1.5	2.2	tr	2.1	2.4
57	1372	1374	*α*-Copaene	13.7	3.1	5.4	0.7	0.2	1.5	1.7	0.8	1.4	0.9	0.8
58	1377	1379	Geranyl acetate						3.5	2.7	4.2	4.0	3.8	4.6
59	1384	1387	*β*-Bourbonene	0.7	0.5	0.5	0.5	0.2	0.1		0.1	0.1		0.2
60	1386	1387	*β*-Cubebene	4.4	0.2	0.4	0.2	0.1	0.4	0.6	0.2		0.2	
61	1388	1389	*β*-Elemene	1.1	2.2	3.1	0.5	0.2	0.4	0.4	0.2	0.3		0.1
62	1402	1409	*α*-Gurjunene		1.6	2.4	0.4							
63	1412	1417	(E)-Caryophyllene	30.6	2.9	3.0	4.7	59.6	4.5	6.3	1.8	3.2	2.7	1.8
64	1424	1430	*β*-Copaene	0.2		0.1	0.2	0.1						
65	1426	1431	*β*-Gurjunene (Calarene)						0.1		0.1			0.1
66	1429	1432	*α*-*trans*-Bergamotene						0.2					
67	1435	1439	Aromadendrene		0.3	0.5	0.3					0.1		0.1
68	1438	1440	(Z)-*β*-Farnesene			0.2								
69	1440	1442	Guaia-6,9-diene		0.4	0.4		0.3						
70	1447	1452	*α*-Humulene	6.4	0.6	0.5	0.4	4.8	0.2	0.3	0.1	0.2		0.1
71	1449	1453	Geranyl acetone		0.2	0.2	0.1							
72	1452	1454	(E)-*β*-Farnesene		0.5		0.1	5.4			tr	tr		
73	1455	1458	Alloaromadendrene	tr		1.1		0.1			tr	tr		
74	1476	1480	Germacrene D	20.0	13.6	20.0	15.9	6.2	14.7	14.7	7.6	14.2	5.8	5.1
75	1479	1482	epi-Bicyclosesquiphellandrene								0.1	0.1		
76	1484	1487	(E)-*β*-Ionone		0.3	0.4								
77	1488	1490	Phenyl ethyl 3-methyl butanoate				0.2							
78	1492	1496	Valencene									0.4	0.7	0.1
79	1494	1496	Viridiflorene (Ledene)					0.1						
80	1498	1500	Bicyclogermacrene	0.6	0.5	1.0	1.6	1.4	2.7	0.9	0.5	0.6	1.0	0.2
81	1499	1500	*α*-Muurolene				0.1	tr						
82	1502	1505	*β*-Bisabolene			0.1								
83	1503	1505	(E,E)-*α*-Farnesene								0.3	0.4		
84	1504	1508	Germacrene A	0.5	0.7	1.1	0.2	0.1	0.2	0.2	0.1	0.2		0.1
85	1510	1513	*γ*-Cadinene				0.3	0.1	0.3					0.3
86	1512	1514	Cubebol	0.6										
87	1521	1522	*δ*-Cadinene	3.6	3.7	5.9	0.9	0.2	0.5	0.6	0.3	0.5	0.3	0.3
88	1523	1528	*cis*-Calamenene			0.1								
89	1541	1544	*α*-Calacorene	tr	tr	0.5	0.1							
90	1546	1548	Elemol								0.1	0.1		0.1
91	1550	1557	1,5-Epoxysalvial-4(14)-ene				0.4		0.2	0.2	0.1	0.1	0.3	0.3
92	1554	1559	Germacrene B			0.1								
93	1557	1561	(E)-Nerolidol								tr			tr
94	1569	1573	epi-Globulol		0.8	1.2								
95	1571	1574	Germacrene D-4-ol	0.5										
96	1574	1577	Spathulenol		0.4	1.0	5.3		1.5	0.6	0.4	0.4	1.6	0.4
97	1579	1582	Caryophyllene oxide	8.4	3.3	4.4	3.3	11.2	1.6	2.3	0.5	0.7	3.6	2.1
98	1585	1590	Globulol			0.4								
99	1588	1590	*β*-Copaen-4-α-ol	0.4		0.4								0.1
100	1590	1594	Salvial-4(14)-en-1-one	0.6			1.1	0.1	0.4	0.3	0.3	0.3	0.3	0.3
101	1598	1600	*n*-Hexadecane		0.1									
102	1601	1602	Ledol				0.4							
103	1603	1604	(2R,5E)-Caryophyll-5-en-12-al			0.4					0.5	0.1		0.2
104	1604	1607	*β*-Oplopenone	0.2						0.2	0.3		0.2	0.1
105	1605	1608	Humulene epoxide II	1.3	0.3	0.3	0.5	0.7	0.2		0.2	0.1		
106	1617	1622	10-epi-*γ*-Eudesmol						0.1		0.1	0.1		0.1
107	1625	1626	(2S,5E)-Caryophyll-5-en-12-al									0.2		0.5
108	1626	1627	1-epi-Cubenol	0.3										
109	1627	1628	Isospathulenol					0.4	0.3				0.2	
110	1629	1631	Allo-aromadendrene epoxide					0.5						
111	1635	1638	epi-*α*-Cadinol			0.2			0.3					
112	1637	1639	Caryophylla-4(12),8(13)-dien-5α-ol or Caryophylla-4(12),8(13)-dien-5β-ol				0.2	0.1			0.7			
113	1639	1640	epi-*α*-Muurolol (tau muurolol)	0.4			0.7			0.3	0.2			
114	1641	1644	*α*-Muurolol									0.1		
115	1644	1649	*β*-Selinene					0.2	0.1		0.1			0.1
116	1645	1649	*β*-Eudesmol						1.1	1.0	1.0	0.6	0.5	0.9
117	1648	1650	Caryophyllenol-II											0.2
118	1649	1652	*α*-Cadinol	0.4	tr	0.2	1.2		0.3	0.3		0.7		
119	1651	1652	*α*-Eudesmol						0.8	0.7	0.7	1.2		0.7
120	1654	1656	Valerianol	0.5			0.7							
121	1657	1661	14-Hydroxy-9-epi-(E)-caryophyllene					0.6						
122	1664	1666	14-Hydroxy-(Z)-caryophyllene			0.2	0.6							
123	1680	1685	Germacra-4(15),5,10(14)-trien-1α-ol			0.2								
124	1683	1685	*α*-Bisabolol									0.1		
125	1684	1688	2,3-Dihydro-farnesol									tr		
126	1688	1690	Endo-8-hydroxy-cycloisolongifolene	0.3					0.2					
127	1690	1691	Vulgarol B							0.3	0.2		0.3	
128	1695	1697	2-Pentadecanone		1.4	1.1								
129	1697	1698	(2Z,6Z)-Farnesol									0.1		
130	1699	1704	*δ*-Dodecalactone								0.2	0.4		0.2
131	1710	1711	Valerenol						0.4	0.2	0.2		0.4	0.3
132	1740	1743	Aromadendrene epoxide									tr		0.1
133	1764	1765	*β*-Costol				0.1							
134	1766	1768	*β*-Bisabolenal				0.2							
135	1778	1779	14-Hydroxy-α-muurolene				0.1							
136	1799	1800	*n*-Octadecane			0.2								
137	1800	1803	14-Hydroxy-*δ*-cadinene				0.1							
138	1820	1826	8,13-Epoxy-15,16-dinorlab-12-ene (sclareol oxide)	0.2			1.3	0.1	1.6	1.5	1.0	0.9	1.9	1.4
139	1842	1845	6,10,14-Trimethyl-2-pentadecanone (Hexahydrofarnesyl acetone)		1.1	1.1								
140	1914		*β*-Springene				0.4		0.3		1.5			
141	1935	1942	Phytol	0.6	0.2	0.2		0.1	0.3			0.1		0.1
142	1974		Ledene oxide-(I)									0.1		0.2
143	1985	1987	Manool oxide				0.4		0.2	0.3	0.3	0.3	0.2	0.3
144	1999	2000	*n*-Eicosane			0.1								
145	2002	2009	13-epi-Manool oxide				0.1		0.1	0.2	0.1	0.2		0.2
146	2023	2026	(E,E)-Geranyl linalool								0.2	0.2		tr
147	2050	2056	Manool				0.6		0.8		0.9	0.6		0.9
148	2055	2059	13-epi-Manool							1.0			0.7	
149	2075	2077	*n*-Octadecanol		3.4	1.4								
150	2099	2100	*n*-Heneicosane		0.2	0.1								
151	2143	2149	Abienol				0.1				0.1	0.2		0.1
152	2200	2200	*n*-Docosane		0.1									
153	2215	2218	(E)-Phytol acetate			0.2								
154	2220	2222	Sclareol				5.1		7.6	10.8	9.8	8.6	10.3	11.2
155	2299	2300	*n*-Tricosane			0.2								
156	2399	2400	*n*-Tetracosane		20.0									
157	2500	2500	*n*-Pentacosane		8.8	0.4								
158	2699	2700	Heptacosane		0.7	0.6								
			Total	98.1	95.9	99.1	84.4	94.4	94.6	95.8	93.6	92.1	98.7	98.1
**Grouped components**				**saeth**	**sargc1**	**sarg2**	**scad**	**sted**	**sscl1**	**sscl2**	**sscl3**	**sscl4**	**sscl5**	**sscl6**
Monoterpene Hydrocarbons				0.9	11.5	15.9	28.0	0.3	3.0	2.8	4.8	3.6	3.8	4.7
Oxygenated Monoterpenes				0.3	9.1	20.3	6.1	0.1	47.1	47.1	56.9	50.0	62.8	62.8
Sesquiterpene Hydrocarbons				82.2	30.8	46.4	27.1	80.2	26.1	25.7	12.3	21.9	11.6	9.6
Oxygenated Sesquiterpenes				13.4	4.8	8.9	14.9	13.7	7.4	6.4	5.5	5.0	7.2	6.6
Diterpenes							0.4		0.3		1.5			
Oxygenated diterpenes				1.3	0.2	0.4	7.6	0.1	10.6	13.8	12.4	11.1	13.1	14.2
Hydrocarbons–Alcohols					3.4	1.4			0.1			0.1		
Hydrocarbons–Aldehydes					3.4	1.6	0.1	tr						
Hydrocarbons–Ketones					2.5	2.2								
Hydrocarbons–Alkanes					29.9	1.6								
Esters							0.2							
Phenylpropanoids												tr		
Others					0.3	0.4					0.2	0.4		0.2
Total				98.1	95.9	99.1	84.4	94.4	94.6	95.8	93.6	92.1	98.7	98.1

RIc = calculated retention indices using an n-alkane standard solution C9–C24 in HP-5 MS column; RI_L_ = literature retention indices; tr = traces (% <0.05).

**Table 2:** Chemical composition of the studied *Salvia* essential oils belonging to Sect. *Eusphace*/I-D clade (src1: cultivated *S. ringens*; sr2-4: wild-growing *S. ringens*).

No	RI_C_	RI_L_	Compounds	src1	sr2	sr3	sr4
				Percentage (%)			
1	920	921	Tricyclene	0.8	0.6	0.6	1.1
2	924	924	*α*-Thujene	0.2		0.6	
3	930	932	*α*-Pinene	9.1	12.5	11.2	11.6
4	944	946	Camphene	15.6	5.5	11.1	10.3
5	968	969	Sabinene	0.1		1.4	
6	972	974	*β*-Pinene	4.8	6.6	7.7	5.5
7	975	977	1-Octen-3-ol		0.1		0.2
8	988	988	Myrcene	2.2	5.7	1.2	2.5
9	1001	1002	*α*-Phellandrene	0.2	0.3		0.1
10	1013	1014	*α*-Terpinene	0.2	0.2	0.4	0.1
11	1025	1026	1,8-Cineole	17.9	34.4	40.2	20.5
12	1029	1031	Benzene acetaldehyde				tr
13	1032	1032	(Z)-*β*-Ocimene			0.3	
14	1043	1044	(E)-*β*-Ocimene				tr
15	1052	1054	*γ*-Terpinene	tr	0.1	0.4	0.1
16	1063	1065	*cis*-Sabinene hydrate	0.1	tr		tr
17	1083	1086	Terpinolene		0.1		0.1
18	1087	1089	*p*-Cymenene	2.4		1.8	
19	1095	1095	Linalool	tr	0.4	1.0	0.5
20	1096	1098	trans-Sabinene hydrate		tr		tr
21	1099	1101	*cis*-Thujone			1.4	
22	1110	1112	*trans*-Thujone				0.3
23	1113	1114	endo-Fenchol		0.1		
24	1115	1118	*cis-p*-Menth-2-en-1-ol		0.9	0.2	0.5
25	1119	1122	*α*-Campholenal		0.1		
26	1135	1137	*trans*-Sabinol	1.0	0.2		
27	1139	1141	Camphor	1.8	1.8	4.5	2.7
28	1143	1145	Camphene hydrate		0.1		0.1
29	1156	1160	Pinocarvone		0.2		tr
30	1161	1165	Borneol	12.9	5.5	1.6	12.7
31	1167	1170	*cis*-Linalool oxide		tr		tr
32	1171	1174	Terpinen-4-ol	1.2	1.5	0.8	1.2
33	1178	1179	*p*-Cymen-8-ol				tr
34	1184	1186	*α*-Terpineol	1.5	0.5	0.8	0.5
35	1189	1194	Myrtenol				0.2
36	1192	1195	Myrtenal		0.1		0.2
37	1193	1195	*cis*-Piperitol	0.4	0.4		
38	1205	1207	*trans*-Piperitol	0.6	0.4		0.2
39	1211	1215	*trans*-Carveol		0.1		0.1
40	1222	1226	*cis*-Carveol		tr		tr
41	1223	1227	Nerol				tr
42	1230	1235	Isobornyl formate				0.1
43	1234	1239	Carvone		tr		
44	1243	1249	Piperitone		0.1		0.1
45	1250	1254	Linalool acetate		0.1		0.1
46	1279	1284	Bornyl acetate	20.3	3.0	2.0	7.9
47	1289	1289	Thymol		0.1		0.1
48	1295	1298	Carvacrol	0.4	0.1		0.1
49	1320	1324	Myrtenyl acetate		tr		
50	1332	1339	*trans*-Carvyl acetate				tr
51	1350	1356	Eugenol				0.9
52	1355	1357	4aα,7α,7aα-Nepetalactone				tr
53	1359	1359	Neryl acetate		tr		
54	1367	1373	*α*-Ylangene		0.1		0.1
55	1372	1374	*α*-Copaene	0.1	0.5	0.3	0.2
56	1377	1379	Geranyl acetate		0.1		0.1
57	1384	1387	*β*-Bourbonene	0.3	0.1	0.5	0.1
58	1399	1402	1,7-di-epi-α-Cedrene				tr
59	1402	1409	*α*-Gurjunene		0.1		0.2
60	1408	1410	*α*-Cedrene				0.2
61	1412	1417	(E)-Caryophyllene	0.8	1.1	2.7	0.7
62	1424	1430	*β*-Copaene		0.1		0.1
63	1426	1431	*β*-Gurjunene (Calarene)				0.1
64	1429	1432	*α*-trans-Bergamotene				0.6
65	1435	1439	Aromadendrene		0.2		
66	1447	1452	*α*-Humulene	0.4	0.3	1.4	0.9
67	1449	1453	Geranyl acetone				tr
68	1452	1454	(E)-*β*-Farnesene		tr		0.2
69	1455	1458	Alloaromadendrene		0.1		0.1
70	1464	1469	*β*-Acoradiene				0.2
71	1472	1478	*γ*-Muurolene		0.4	0.7	0.4
72	1475	1479	ar-Curcumene				1.4
73	1476	1480	Germacrene D			0.9	
74	1478	1481	*γ*-Curcumene				0.1
75	1480	1483	*α*-Amorphene	0.4	0.1		0.2
76	1492	1496	Valencene		0.2		
77	1499	1500	*α*-Muurolene	0.1	0.1	0.2	0.2
78	1502	1505	*β*-Bisabolene				0.3
79	1510	1513	*γ*-Cadinene	0.3	0.2	0.3	0.4
80	1520	1521	*trans*-Calamenene		0.3	0.7	
81	1521	1522	*δ*-Cadinene	0.3	0.1	0.6	0.5
82	1523	1528	*cis*-Calamenene	0.2			0.3
83	1531	1537	*α*-Cadinene		tr		0.1
84	1539	1542	*cis*-Sesquisabinene hydrate				0.3
85	1541	1544	*α*-Calacorene		0.1		0.1
86	1569	1573	epi-Globulol		0.1		
87	1571	1574	Germacrene D-4-ol	0.2			
88	1574	1577	Spathulenol		0.7		1.1
89	1579	1582	Caryophyllene oxide	1.4	2.0	1.2	0.7
90	1580	1586	Gleenol		0.1		0.1
91	1589	1592	Viridiflorol		0.9		0.2
92	1597	1600	Rosifoliol				0.2
93	1601	1602	Ledol				0.3
94	1605	1608	Humulene epoxide II	0.8	0.5	0.5	1.4
95	1615	1618	1,10-di-epi-Cubenol				0.2
96	1626	1627	1-epi-Cubenol				0.2
97	1630	1632	*α*-Acorenol				0.2
98	1635	1638	epi-α-Cadinol		0.4		
99	1637	1639	Caryophylla-4(12),8(13)-dien-5α-ol or Caryophylla-4(12),8(13)-dien-5β-ol		0.2		0.4
100	1639	1640	epi-α-Muurolol (tau muurolol)		0.1		0.3
101	1641	1644	*α*-Muurolol				0.5
102	1644	1649	*β*-Selinene		0.1		
103	1649	1652	*α*-Cadinol	0.6	0.3	0.2	0.6
104	1665	1670	epi-β-Bisabolol				1.6
105	1671	1674	Valeranone		6.4		
106	1683	1685	*α*-Bisabolol		0.3		0.5
107	1985	1987	Manool oxide		tr		0.1
108	2002	2009	13-epi-Manool oxide		tr		0.1
109	2025	2030	(6Z,10E)-Pseudo phytol				tr
110	2050	2056	Manool				tr
111	2053	2058	(6Ε,10E)-Pseudo phytol		tr		
112	2220	2222	Sclareol		tr		0.1
			Total	99.6	98.1	99.4	97.5
**Grouped components**				**src1**	**sr2**	**sr3**	**sr4**
Monoterpene Hydrocarbons				35.6	31.6	36.7	31.4
Oxygenated Monoterpenes				58.1	50.2	52.5	48.2
Sesquiterpene Hydrocarbons				2.9	4.2	8.3	7.7
Oxygenated Sesquiterpenes				3.0	12.0	1.9	8.8
Oxygenated diterpenes							0.3
Hydrocarbons–Alcohols					0.1		0.2
Hydrocarbons–Aldehydes							tr
Phenylpropanoids							0.9
Total				99.6	98.1	99.4	97.5

RIc = calculated retention indices using an n-alkane standard solution C9–C24 in HP-5 MS column; RI_L_ = literature retention indices; tr = traces (% <0.05).

**Table 3:** Chemical composition of the studied *Salvia* essential oils belonging to Sect. *Hemisphace*/I-B clade (sverc1: cultivated *S. verticillata*; sver2: wild-growing *S. verticillata*).

No	RI_C_	RI_L_	Compounds	sverc1	sver2
				Percentage (%)	
1	930	932	*α*-Pinene	1.0	0.6
2	944	946	Camphene		tr
3	960	961	Verbenene	0.2	
4	968	969	Sabinene	0.2	0.6
5	972	974	*β*-Pinene	6.1	1.6
6	988	988	Myrcene	0.3	0.2
7	1013	1014	*α*-Terpinene		0.1
8	1023	1024	Limonene	1.8	
9	1025	1026	1,8-Cineole		18.4
10	1032	1032	(Z)-*β*-Ocimene		0.7
11	1043	1044	(E)-*β*-Ocimene		1.3
12	1052	1054	*γ*-Terpinene		0.7
13	1063	1065	*cis*-Sabinene hydrate		0.1
14	1083	1086	Terpinolene		tr
15	1095	1095	Linalool	0.2	0.1
16	1098	1100	*n*-Nonanal	0.4	
17	1099	1101	*cis*-Thujone		0.1
18	1119	1122	*α*-Campholenal		0.1
19	1131	1135	*trans*-Pinocarveol		0.1
20	1134	1137	*cis*-Verbenol	0.4	0.1
21	1139	1141	Camphor		0.1
22	1156	1160	Pinocarvone		0.1
23	1171	1174	Terpinen-4-ol		0.2
24	1184	1186	*α*-Terpineol		1.2
25	1189	1194	Myrtenol		0.1
26	1192	1195	Myrtenal		0.2
27	1198	1200	*trans*-Dihydrocarvone		tr
28	1295	1298	Carvacrol		0.1
29	1300	1300	*n*-Tridecane	0.3	
30	1342	1348	*α*-Cubebene	0.4	
31	1355	1357	4aα,7α,7aα-Nepetalactone		51.4
32	1372	1374	*α*-Copaene	2.3	0.2
33	1380	1386	4aα,7α,7aβ-Nepetalactone		1.2
34	1384	1387	*β*-Bourbonene	3.0	1.3
35	1386	1387	*β*-Cubebene	0.8	
36	1388	1389	*β*-Elemene	0.7	0.2
37	1389	1391	4a-α,7β,7aα-Nepetalactone		0.6
38	1402	1409	*α*-Gurjunene	0.4	
39	1412	1417	(E)-Caryophyllene	3.8	1.3
40	1424	1430	*β*-Copaene		0.1
41	1426	1431	*β*-Gurjunene (Calarene)	2.0	
42	1447	1452	*α*-Humulene	0.7	0.4
43	1452	1454	(E)-*β*-Farnesene		1.5
44	1455	1458	Alloaromadendrene	0.4	
45	1472	1478	*γ*-Muurolene	1.2	
46	1476	1480	Germacrene D	11.5	2.8
47	1479	1482	epi-Bicyclosesquiphellandrene	0.4	
48	1480	1483	*α*-Amorphene	0.4	
49	1498	1500	Bicyclogermacrene		0.1
50	1499	1500	*α*-Muurolene	0.1	
51	1502	1505	*β*-Bisabolene		0.1
52	1504	1508	Germacrene A		0.1
53	1510	1513	*γ*-Cadinene	0.8	
54	1521	1522	*δ*-Cadinene	1.2	0.2
55	1541	1544	*α*-Calacorene		tr
56	1550	1557	1,5-Epoxy-salvial(4)14-ene		0.1
57	1557	1561	(E)-Nerolidol	35.0	
58	1574	1577	Spathulenol	1.1	0.1
59	1579	1582	Caryophyllene oxide	2.6	6.0
60	1585	1590	Globulol	1.6	
61	1588	1590	*β*-Copaen-4α-ol	1.0	0.1
62	1589	1592	Viridiflorol		0.2
63	1590	1594	Salvial-4(14)-en-1-one	1.8	0.2
64	1605	1608	Humulene epoxide II	0.4	1.0
65	1629	1631	Allo-aromadendrene epoxide	0.5	
66	1635	1638	epi-α-Cadinol	0.6	0.3
67	1637	1639	Caryophylla-4(12),8(13)-dien-5α-ol or Caryophylla-4(12),8(13)-dien-5β-ol	0.2	0.2
68	1639	1640	epi-α-Muurolol (tau muurolol)	2.2	0.4
69	1649	1652	*α*-Cadinol	0.3	0.1
70	1654	1656	Valerianol	0.5	
71	1656	1660	*cis*-Calamenen-10-ol		0.1
72	1657	1661	14-Hydroxy-9-epi-(E)-caryophyllene	0.4	0.1
73	1664	1666	14-Hydroxy-(Z)-caryophyllene	0.7	
74	1710	1711	Valerenol	1.6	0.1
75	1800	1803	14-Hydroxy-δ-cadinene		0.1
76	1842	1845	6,10,14-Trimethyl-2-pentadecanone (hexahydrofarnesyl acetone)	0.7	
77	1935	1942	Phytol		tr
78	2399	2400	*n*-Tetracosane		tr
79	2500	2500	*n*-Pentacosane	0.2	tr
			Total	92.4	97.4
**Grouped components**				**sverc1**	**sver2**
Monoterpene Hydrocarbons				9.6	5.8
Oxygenated Monoterpenes				0.6	74.2
Sesquiterpene Hydrocarbons				30.1	8.3
Oxygenated Sesquiterpenes				50.5	9.1
Oxygenated diterpenes					tr
Hydrocarbons–Aldehydes				0.4	
Hydrocarbons–Ketones				0.7	
Alkanes				0.5	tr
Total				92.4	97.4

RIc = calculated retention indices using an n-alkane standard solution C9–C24 in HP-5 MS column; RI_L_ = literature retention indices; tr = traces (% <0.05).

**Table 4:** Chemical composition of the studied *Salvia* essential oils belonging to Sect. *Plethiosphace*/I-C clade (samp1–5: wild-growing *S. amplexicaulis*; sampc6: cultivated *S. amplexicaulis*; sprc: cultivated *S. pratensis* subsp. *pratensis*; spr: wild-growing *S. pratensis* subsp. *pratensis*; svirg: wild-growing *S. virgata*).

No	RI_C_	RI_L_	Compounds	samp1	samp2	samp3	samp4	samp5	sampc6	sprc1	spr2	svirg
				Percentage (%)								
1	920	921	Tricyclene						0.6			
2	924	924	*α*-Thujene							0.9	2.3	2.6
3	930	932	*α*-Pinene	0.2	0.2	0.1	0.2	0.2	14.4	0.1	1.6	1.9
4	944	946	Camphene						12.9		1.8	2.2
5	968	969	Sabinene	0.5	0.1	0.1		tr	tr	14.8	15.0	21.2
6	972	974	*β*-Pinene						8.6		1.8	1.3
7	975	977	1-Octen-3-ol			0.2	0.6	1.1				
8	988	988	Myrcene	0.8	0.3				1.8	0.4		0.8
9	1013	1014	*α*-Terpinene							0.4	1.0	0.9
10	1023	1024	Limonene							0.2		0.8
11	1025	1026	1,8-Cineole	0.4		0.1	0.1	0.4	19.6		1.7	
12	1052	1054	*γ*-Terpinene	0.3		tr				0.8	3.3	1.9
13	1063	1065	*cis*-Sabinene hydrate							0.1	0.5	0.2
14	1083	1086	Terpinolene							0.1		0.3
15	1087	1089	*p*-Cymenene	0.3		tr			1.3	0.4	0.4	0.2
16	1095	1095	Linalool		0.1		0.1	tr				
17	1096	1098	*trans*-Sabinene hydrate			tr				0.1	0.3	
18	1098	1100	*n*-Nonanal		0.1		0.2			0.1		0.1
19	1139	1141	Camphor						0.5			
20	1161	1165	Borneol						0.4		2.7	0.3
21	1171	1174	Terpinen-4-ol	0.3		tr				0.4	1.5	0.6
22	1184	1186	*α*-Terpineol				tr					
23	1198	1200	*trans*-Dihydrocarvone				tr					
24	1200	1201	*n*-Decanal				0.1					
25	1250	1254	Linalool acetate				tr	tr				
26	1279	1284	Bornyl acetate						0.6			3.7
27	1295	1298	Carvacrol			0.1	tr			0.3		
28	1342	1348	*α*-Cubebene	0.3	0.2	0.1	0.4	tr				0.2
29	1355	1357	4aα,7α,7aα-Nepetalactone									0.6
30	1367	1373	*α*-Ylangene	tr	0.1		0.2	tr				
31	1372	1374	*α*-Copaene	0.9	0.6	tr	0.9	0.9	0.4			0.4
32	1384	1387	*β*-Bourbonene	1.0	0.7	0.3	0.9	1.3	0.4			
33	1386	1387	*β*-Cubebene	tr	0.2	0.3	0.3	tr				0.5
34	1388	1389	*β*-Elemene	0.3	0.5	1.3	0.7	1.1				0.3
35	1412	1417	(E)-Caryophyllene	5.7	6.5	7.2	6.7	14.8	1.2	45.5	46.9	20.8
36	1424	1430	*β*-Copaene	0.4	0.4	0.3	0.6	0.7				
37	1426	1431	*β*-Gurjunene (Calarene)			0.4						0.3
38	1435	1439	Aromadendrene		0.1						1.1	
39	1447	1452	*α*-Humulene	0.8	0.4	0.9	0.9	1.7	tr	2.2	1.4	1.1
40	1449	1453	Geranyl acetone	0.2	0.2							
41	1452	1454	(E)-*β*-Farnesene	0.5	1.0	0.8	1.3	2.0	tr			0.5
42	1455	1458	Alloaromadendrene	tr	0.4	2.7	5.1	4.7	3.8			15.2
43	1472	1478	*γ*-Muurolene	1.7					tr			
44	1476	1480	Germacrene D	4.0	10.0	39.0	20.1	40.2	tr		2.9	6.1
45	1480	1483	*α*-Amorphene	0.8			1.5	1.2	0.8			
46	1484	1487	(E)-*β*-Ionone	1.0	0.2	0.7			1.9	0.1		0.2
47	1498	1500	Bicyclogermacrene		1.3	1.9				0.4		0.6
48	1499	1500	*α*-Muurolene	0.3	0.2		0.4	0.6				
49	1503	1505	(E,E)-*α*-Farnesene							0.7		
50	1504	1508	Germacrene A		0.1	0.8	0.2					
51	1510	1513	*γ*-Cadinene	1.1	0.8	0.9	1.6	1.0	0.5			
52	1521	1522	*δ*-Cadinene	1.3	1.4	1.5	2.4	2.0	0.5			0.3
53	1529	1531	(Z)-Nerolidol		0.2							
54	1531	1537	*α*-Cadinene				0.2					
55	1541	1544	*α*-Calacorene	tr	0.2		0.2					
56	1550	1557	1,5-Epoxy-salvial(4)14-ene	0.7	0.6	0.5	0.5		0.6			
57	1553	1559	Germacrene B								1.3	
58	1557	1561	(E)-Nerolidol	0.3	0.7		0.4					3.7
59	1574	1577	Spathulenol	0.7	18.7	2.4	1.8	0.7	0.4			0.6
60	1579	1582	Caryophyllene oxide	35.1	10.0	8.0	7.5	6.8	5.2	25.8	10.4	6.6
61	1589	1592	Viridiflorol		0.6	0.4	12.1					0.6
62	1590	1594	Salvial-4(14)-en-1-one	7.0	5.8	3.4		3.7	3.7			0.4
63	1604	1607	*β*-Oplopenone	4.4	3.4				1.8			
64	1605	1608	Humulene epoxide II	3.6		1.3	1.4	1.0		0.9		0.3
65	1627	1628	Isospathulenol							2.0		0.2
66	1629	1631	Allo-aromadendrene epoxide				2.3					
67	1635	1638	epi-*α*-Cadinol		1.1	1.1	1.0					0.3
68	1637	1639	Caryophylla-4(12),8(13)-dien-5α-ol or Caryophylla-4(12),8(13)-dien-5β-ol	0.4	1.0		1.2			0.3		
69	1639	1640	epi-*α*-Muurolol (tau muurolol)	1.0				0.8				
70	1640	1643	Caryophylla-3,8(15)-dien-5α-ol				5.2	0.5				
71	1641	1644	*α*-Muurolol		2.1							
72	1644	1649	*β*-Selinene		0.3		0.6					
73	1648	1650	Caryophyllenol-II	3.4			0.9					
74	1649	1652	*α*-Cadinol	0.7	0.9	2.4	1.3	4.4				
75	1656	1660	*cis*-Calamenen-10-ol				0.4					
76	1666	1668	*trans*-Calamenen-10-ol				0.5					
77	1685	1687	Eudesma-4(15),7-dien-1β-ol		1.3							
78	1688	1690	Endo-8-hydroxy-cycloisolongifolene					4.0				
79	1690	1691	Vulgarol B	6.4	0.6	1.1	4.4		2.9			
80	1691	1691	Eudesma-4,11-dien-2-ol		6.3		1.1					
81	1710	1711	Valerenol		1.3	3.7	1.3	1.1	2.0			
82	1740	1743	Aromadendrene epoxide		0.6			0.5				
83	1764	1765	*β*-Costol	0.8	0.5		0.3					
84	1770	1773	*α*-Costol				0.3					
85	1842	1845	6,10,14-Trimethyl-2-pentadecanone (hexahydrofarnesyl acetone)	0.9	0.6		0.6		1.3			0.8
86	1900	1900	*n*-Nonadecane				0.2					
87	1935	1942	Phytol	1.0	0.2	0.1	0.4	0.6	5.5	0.2		
88	1944	1946	Isophytol		0.3							
89	1974		Ledene oxide-(I)		6.3	3.7	1.5	1.6				
90	2299	2300	*n*-Tricosane				0.1					
91	2399	2400	*n*-Tetracosane	0.3			0.1					
92	2500	2500	*n*-Pentacosane	0.9	0.2		0.2					
93	2600	2600	Hexacosane				0.2			0.2		
94	2699	2700	Heptacosane				0.5					
95	2800	2800	Octacosane				0.1					
96	2900	2900	Nonacosane				0.3					
			Total	90.7	89.9	87.8	94.6	99.6	93.6	97.4	97.9	99.6
**Grouped components**				**samp1**	**samp2**	**samp3**	**samp4**	**samp5**	**sampc6**	**sprc1**	**spr2**	**svirg**
Monoterpene Hydrocarbons				2.1	0.6	0.2	0.2	0.2	39.6	18.1	27.2	34.1
Oxygenated Monoterpenes				0.9	0.3	0.2	0.3	0.4	21.1	0.9	6.7	5.4
Sesquiterpene Hydrocarbons				19.1	25.6	58.4	45.2	72.2	7.6	48.8	53.6	46.3
Oxygenated Sesquiterpenes				64.5	61.8	28.0	45.4	25.1	16.6	29.0	10.4	12.7
Oxygenated diterpenes				1.0	0.5	0.1	0.4	0.6	5.5	0.2		
Hydrocarbons–Alcohols						0.2	0.8	1.1				
Hydrocarbons–Aldehydes					0.1		0.2			0.1		0.1
Hydrocarbons–Ketones				0.9	0.6		0.6		1.3			0.8
Alkanes				1.2	0.2		1.5			0.2		
Others				1.0	0.2	0.7			1.9	0.1		0.2
Total				90.7	89.9	87.8	94.6	99.6	93.6	97.4	97.9	99.6

RIc = calculated retention indices using an n-alkane standard solution C9–C24 in HP-5 MS column; RI_L_ = literature retention indices; tr = traces (% <0.05).

## Data Availability

All data are presented in the results of this study (datasets are available upon request).

## Supplementary Materials

The following supporting information can be downloaded at: https://www.mdpi.com/article/10.3390/molecules29071547/s1, Table S1. List of the investigated *Salvia* species in different sections with provenance, abbreviations used (essential oil code, EO), voucher specimens, and/or living material in ex situ conservation at the Balkan Botanic Garden of Kroussia (BBGK) with IPEN (International Plant Exchange Network) code and yield; Table S2. Overview of essential oil compositions of 10 *Salvia* species in different sections based on literature sources; Figure S1. Principal component analysis of the major chemical classes by *Salvia* groups as defined in HCA.
