# Supplementary material for: Chemometric Analysis Evidencing the Variability in the Composition of Essential Oils in 10 Salvia Species from Different Taxonomic Sections or Phylogenetic Clades
Source: Molecules. 2024 Mar 29;29(7):1547. doi: 10.3390/molecules29071547 (PMC11013157; doi:10.3390/molecules29071547)
Supplement: Supplementary file 1 [file molecules-29-01547-s001.zip › molecules-2878230-supplementary.pdf]

Chemometric Analysis Evidencing the Variability in the Composition of Essential Oils in 10 *Salvia* Species from Different Taxonomic Sections or Phylogenetic Clades

Ekaterina-Michaela Tomou[1,*], Panagiota Fraskou[1], Konstantina Dimakopoulou[2], Eleftherios Dariotis[3], Nikos Krigas[3], Helen Skaltsa[1,*]

[1]Department of Pharmacognosy & Chemistry of Natural Products, Faculty of Pharmacy, National and Kapodistrian University of Athens, Panepistimiopolis, Zografou, 15771 Athens, Greece; pfraskou@pharm.uoa.gr (P.F.)

[2]Department of Hygiene, Epidemiology and Medical Statistics, Medical School, National and Kapodistrian University of Athens, 11527 Athens, Greece; kdimakop@med.uoa.gr (K.D.)

[3]Institute of Plant Breeding and Genetic Resources, Hellenic Agricultural Organization DEMETER (ELGO Dimitra), 57001 Thermi, Greece; eleftheriosdariotis@yahoo.com (E.D.); nkrigas@elgo.gr (N.K.)

[*]Correspondence: ktomou@pharm.uoa.gr (E.-M.T.); skaltsa@pharm.uoa.gr; Tel.: + 30-210-727-4593/4301 (H.S.)

Table of contents

**Table S1.** List of the investigated *Salvia* species in different sections with provenance, abbreviations used (essential oil code, EO), voucher specimens and/or living material in ex-situ conservation at the Balkan Botanic Garden of Kroussia (BBGK) with IPEN (International Plant Exchange Network) code, and yield	3, 4
**Table S2.** Overview of essential oil compositions of 10 *Salvia* species in different sections based on literature sources.	5-16
**Figure S1.** Principal Component Analysis of the major chemical classes by *Salvia* groups as defined in HCA.	17

Table S1. List of the investigated *Salvia* species in different sections with provenance, abbreviations used (essential oil code, EO), voucher specimens and/or living material in ex-situ conservation at the Balkan Botanic Garden of Kroussia (BBGK) with IPEN (International Plant Exchange Network) code,and yield.

No	*Salvia* species (type of material)	Collection area-Country	Latitude, Longitude	Altitude (m)	Collection date	Voucher specimen / IPEN BBGK	EO code	Oil yield (%)*
			***Aethiopis* section / I-C clade**					
**1**	***S. aethiopis*** (wild)	Kilkis-Greece	40.983133, 22.862810	200	13/6/2021	SAN-004	saeth	1.00
**2**	***S. argentea*** (cultivated)	Mt Ymittos-Greece	37.9495956, 23.8416775	237	22/5/2021	SAC-001	sargc1	0.60
**3**	***S. argentea*** (wild)	Mt Pateras-Greece	38.122189, 23.309605	600	26/5/2021	SAN-001	sarg2	0.60
**4**	***S. candidissima*** (wild)	Mt Geraneia (Loutraki)-Greece	37.978257, 23.016677	220	20/6/2021	SAN-009	scad	1.5
**5**	***S. sclarea*** (wild)	Agkistro-Greece	41.336902, 23.347520	82	12/6/2021	SAN-005	sscl1	0.90
**6**	***S. sclarea*** (wild)	Mt Lekani (Kirgia, Drama)-Greece	41.105570, 24.298979	200	21/6/2021	SAN-019	sscl3	1.10
**7**	***S. sclarea*** (wild)	Mt Tzena (South)-Greece	41.111465, 22.224022	672	3/7/2021	SAN-016	sscl5	1.00
**8**	***S. sclarea*** (wild)	Vrontero-Greece	40.785645, 21.051008	887	4/7/2021	SAN-017	sscl6	0.90
**9**	***S. sclarea*** (wild)	Mt Voras-Greece	40.882333, 21.910825	1191	3/7/2021	SAN-018	sscl2	1.10
**10**	***S. sclarea*** (wild)	Mt Oiti-Greece	38.7227316, 22.3291290	1120	7/6/2021	SAN-006	sscl4	1.10
**11**	***S. teddii*** (wild)	Mt Karpouzi-Greece	41.1185983, 24.7529153	880	15/6/2021	SAN-007	sted	1.00
			***Eusphace* section / I-D clade**					
**12**	***S. ringens*** (cultivated)	Brusani (Kavadarci)-North Macedonia	41.400922, 21.936606	280	15/6/2021	SAC-005/ RNM-1-BBGK-19,445	src1	0.90

**13**	***S. ringens*** (wild)	Mt Tzena (South)-Greece	41.059250, 22.150830	377	3/7/2021	SAN-014	sr4	0.75
**14**	***S. ringens*** (wild)	Mt Parnassos-Greece	38.141817, 23.732262	680	7/6/2021	SAN-008	sr3	1.00
**15**	***S. ringens*** (wild)	Mt Devas-Greece	40.811775, 21.067413	1051	4/7/2021	SAN-015	sr2	1.10
***Hemisphace* section / I-B clade**								
**16**	***S. verticillata*** (cultivated)	Andritsaina-Greece	37.4932195, 21.8988616	757	31/5/2021	SAC-003	sverc1	0.50
**17**	***S. verticillata*** (wild)	Mt Sfika-Greece	40.677315, 21.092043	1532	4/7/2021	SAN-020	sver2	1.25
***Plethiosphace* section / I-C clade**								
**18**	***S. amplexicaulis*** (wild)	Agkistro-Greece	41.383893, 23.383829	132	12/6/2021	SAN-002	samp1	0.60
**19**	***S. amplexicaulis*** (wild)	Mt Karpouzi-Greece	41.139852, 24.7389694	500	15/6/2021	SAN-003	samp3	0.75
**20**	***S. amplexicaulis*** (wild)	Mt Tzena (South)-Greece	41.108544, 22.220554	626	3/7/2021	SAN-011	samp4	0.55
**21**	***S. amplexicaulis*** (cultivated)	Mt Vertiskos-Greece	40.878056, 23.224167	750	15/6/2021	SAC-002/ GR-1-BBGK-99,1059	sampc6	0.60
**22**	***S. amplexicaulis*** (wild)	Vrontero-Greece	40.785645, 21.051008	887	4/7/2021	SAN-012	samp5	0.60
**23**	***S. amplexicaulis*** (wild)	Mt Voras-Greece	40.879878, 21.898733	1201	3/7/2021	SAN-010	samp2	0.65
**24**	**S. *pratensis* subsp. *pratensis*** (cultivated)	Belchishko Marsh-North Macedonia	41.317911, 20.817275	772	15/6/2021	SAC-004/ RNM-1-BBGK-19,527	sprc1	0.90
**25**	**S. *pratensis* subsp. *pratensis*** (wild)	Vasilitsa (Grevena)-Greece	40.050113, 21.240552	1085	5/7/2021	SAN-013	spr2	1.00
**26**	***S. virgata*** (cultivated)	Mornos Lake-Greece	38.5567136, 22.1760425	460	31/5/2021	SAC-006	svirg	0.75

*The essential-oil yield is given in percentage (% v/w) based on the dry weight of the plant material.

Table S2. Overview of essential oil compositions of 10 *Salvia* species in different sections based on literature sources.

Species	Geographic origin (regions) (type of material / plant parts examined / Other information)	Major compounds (>5%)	Chemical groups	Reference
		Aethiopis section / I-C clade		
Salvia aethiopis	Former Yugoslavia	bornyl acetate (21.8%), unidentified (20.5; 11.0; 5.1%)	n.d.	[29]
	Former Yugoslavia	β-caryophyllene (27.5%), germacrene D (10.9%), caryophyllene oxide (6.4%), α-humulene (5.7%)	n.d.	[30]
	Serbia	β-caryophyllene (36.8%), α-copaene (33.4%), γ-muurolene (10.3%), β-elemene (7.3%), α-muurolene (7.2%)	Sesquiterpene hydrocarbons: 96.9%<br>Monoterpene hydrocarbons: 2.8%	[24]
	Serbia<br>(flower, leaf, stem)	bicyclogermacrene (29.0%, 9.2%, 11.2%), α-copaene (16.3%, 22.4%, 17.0%), spathulenol (14.0%, 14.4%, 20.1%), germacrene D (13.2%, 13.2%, 13.5%), β-cubebene (5.0%,6.3%, 5.8%)	Sesquiterpenoids: 88.8-94.6%,<br>Diterpenoids: 0-0.3%<br>Monoterpenoids: 0.2%	[23]
	Serbia	bicyclogermacrene (31.3%), α-copaene (12.9%), (E)-caryophyllene (11.7%), germacrene D (18.2%)	Sesquiterpenoids: 96.1%<br>Monoterpenoids: 2.3%	[22]
	Spain	α-copaene (9.15%; 9.16%; 10.43%), germacrene D (7.50%; 10.46%; 4.95%), bicyclogermacrene (29.54%; 41.48%; 33.97%)	n.d.	[25]
	Iran	β-caryophyllene (24.6%), α-copaene (15.5%), germacrene D (13.5%), caryophyllene oxide (8.0%), β-elemene (6.0%), α-humulene (5.0%)	Sesquiterpenes: 88.8%<br>Monoterpenes:2.8%	[19]
	Iran	β-caryophyllene (17.0%), α-copaene (16.3%), germacrene D (13.8%), β-cubebene (9.7%), spathulenol (8.3%), δ-cadinene (7.7%), α-humulene (6.9%)	n.d.	[20]

	Iran (leaf, flowers, stem, and aerial parts)	β-caryophyllene (20.9-24.1%), α-copaene (18.1-24.9%), germacrene D (17.8-18.6%), δ-cadinene (6.3-7.0%), β-cubebene (5.9-7.0%), α-humulene (5.4-6.8%)	Sesquiterpenes: 85.7-91.1%<br>Monoterpenes: 1.1-5.6%	[21]
	Iran	linalool (0.6-51.6%), α-copaene (0-24.3%), β-elemene (1.64-9.37%), (E)-caryophyllene (9.0-18.9%), germacrene D (3.96-25.2%), bicyclogermacrene (0.79-5.78%), δ-cadinene (0-8.75%), caryophyllene oxide (0-10.3%)	n.d.	[18]
	Turkey	germacrene D (29.0%), α-copaene (19.8%), β-cubebene+β-elemene (9.9%), bicyclogermacrene (9.3%), δ-cadinene (8.7%), β-caryophyllene (7.3%)	n.d.	[26]
	Turkey	α-copaene (18.21%), α-cubebene (12.36%), spathulenol (12.25%), germacrene D (8.21%), 1,8-cineole (7.17%)	n.d.	[27]
	Turkey	palmitic acid (8.53%)	Oxygenated sesquiterpenes: 26.66%<br>Hydrocarbons and derivatives: 26.41%<br>Fatty acids and derivatives: 11.48%<br>Sesquiterpene hydrocarbons: 7.26%<br>Diterpene alcohols: 6.09%<br>Oxygenated monoterpenes: 4.96%<br>Others: 4.35%<br>Oxygenated triterpenes: 2.47%<br>Monoterpene hydrocarbons: 0.83%<br>Phenolic compounds: 0.51%	[28]
S. argentea	Morocco	camphor (45.1%), camphene (19.4%), α-pinene (9.3%), borneol (9.0%), cis-thujone (7.5%)	Oxygenated monoterpenes: 64.1%<br>Monoterpene hydrocarbons: 28.7%	[32,33]
	Serbia	viridiflorol (32.4%), manool (14.6%), α-humulene (10.7%), cis-thujone (7.3%)	Oxygenated sesquiterpenes: 39.4%<br>Oxygenated monoterpenes: 13.1%<br>Sesquiterpene hydrocarbons: 12.7%	[32,34]

			Carbonylic compounds: 7.7%<br>Hydrocarbons: 4.0%<br>Monoterpene hydrocarbons: 0.5%	
	Tunisia (Sers; Makther)	viridiflorol (26.93; 18.75%), manool (6.15; 13.59%), p-cymene (4.17; 6.39%), α-humulene (4.13; 5.28%), cis-thujone (7.30; 8.06%)	Oxygenated sesquiterpenes: 36.65; 29.71%<br>Oxygenated monoterpenes: 22.18; 20.65%<br>Monoterpene hydrocarbons: 14.55; 13.43%<br>Sesquiterpene hydrocarbons: 14.27; 10.05%<br>Oxygenated diterpenes: 6.15; 13.59%<br>Others: 0.55; 0.36%	[36]
	Tunisia	manool (1.5-20.15%), manool oxide (1.12-18.1%), viridiflorol (15.9-4.7%), camphor (3.79-9.02%), τ-cadinol (1.35-7.0%), methyl eugenol (0.53-6.87%), β-ionone (2.86-6.57%), 1,8-cineole (2.8-5.8%), α-cadinol (1.58-5.29%)	Oxygenated sesquiterpenes: 24.84-33.25%,<br>Oxygenated monoterpenes: 12.02-21.0%,<br>Monoterpene hydrocarbons: 1.90-3.15%<br>Sesquiterpene hydrocarbons: 4.76-13.26%<br>Diterpenes: 4.64-38.25%<br>Others: 9.23-21.45%	[37]
	North Macedonia	caryophyllene oxide (37.6%), α-copaene (8.5%), humulene epoxide II (6.3%), β-caryophyllene (6.1%)	Oxygenated sesquiterpenes: 51.0%<br>Sesquiterpene hydrocarbons: 30.2%<br>Hydrocarbons: 9.4%<br>Carbonylic compounds: 5.4% Others: 3.1%<br>Oxygenated monoterpenes: 0.6%	[32,35]
	Italy	14-hydroxy-α-humulene (40.1%), 1,3,8-p-menthatriene (12.1%), globulol (7.4%), β-sesquiphellandrene (5.8%)	Oxygenated sesquiterpenes: 58.6%<br>Monoterpene hydrocarbons: 21.4%<br>Sesquiterpene hydrocarbons: 13.6%<br>Hydrocarbons: 0.1%<br>Carbonyl compounds: 0.1%	[32]
	Greece	germacrene-D (37.41%), β-caryophyllene (6.75%)	n.d.	[31]
	Turkey	sclareol (40.01%), germacrene D (13.90%), β-pinene (11.93%), sclareol oxide (9.65%), α-pinene (6.59%)	Monoterpene hydrocarbons: 24.26%	[38]

			Sesquiterpene hydrocarbons: 18.40%<br>Oxygenated diterpenes: 49.66%	
S. candidissima	Greece	α-pinene (11.2%), 1,8-cineole (9.9%), p-cymene (7.4%), myrtenal (6.5%), pinocarvone (6.2%), camphene (5.7%), trans-pinocarveol (5.5%)	Monoterpenes: 70.9%<br>Sesquiterpenes: 12.5%<br>Diterpenes: 5.6%	[39]
	Turkey	β-pinene (34.4%), α-pinene (22.6%), 1,8-cineole (8.4%)	n.d.	[40]
	Turkey	camphor (28.94%), bornyl acetate (12.80%), borneol (9.44%), β-cadinene (5.88%), α-caryophyllene (5.40%), 1,8-cineole (5.15%)	Oxygenated monoterpenes: 61.08%<br>Monoterpene hydrocarbons: 15.87%<br>Sesquiterpene hydrocarbons: 15.14%<br>Oxygenated sesquiterpenes: 7.56%	[41]
	Turkey	spathulenol (12.75%), caryophyllene oxide (8.67%), ledene oxide (6.98%), o-cymene (6.03%)	n.d.	[42]
S. sclarea	Egypt<br>(cultivated; leaves, flowers)	sclareoloxide (27.3; 6.8%), thymol (20.6; 4.2%), linalyl acetate (2.2; 10.6%), caryophyllene oxide (9.9; 2.7%), manoyl oxide (1.1; 9.6%), linalool (0; 6.5%)	Oxygenated sesquiterpenes: 50.6; 14.9%,<br>Oxygenated monoterpenes: 27.0; 24.8%,<br>Oxygenated diterpenes: 7.2; 48.3%<br>Monoterpene hydrocarbons: 1.3; 0.3%	[43]
	Southern France	linalyl acetate (81.1%), linalool (10.7%)	n.d.	[44]
	Germany<br>(cultivated)	linalyl acetate (36.33%), linalool (23.47%), sclareol (14.62%), α-terpineol (8.12%)	n.d.	[45]
	Greece	linalool (17.2%), linalyl acetate (14.3%), geraniol (6.5%), geranyl acetate (7.5%), terpineol (15.1%), nerol (5.5%), neryl acetate (5.2%), sclareol (5.2%)	n.d.	[46]
	Greece<br>(two localities: central; northwestern Greece)	linalyl acetate (19.75; 31.05%), linalool (30.43; 18.46%), α-terpineol (5.08; 7.56%), geranyl acetate (12.1; 4.45%), sclareol (3.53; 5.55%)	Oxygenated monoterpenes: 64.77-80.79%<br>Sesquiterpene hydrocarbons: 4.91-5.80%<br>Diterpenes: 4.22-11.43%<br>Monoterpene hydrocarbons: 3.74-5.03%<br>Oxygenated sesquiterpenes: 2.89-6.23%	[47]

	Greece (hydrodistillation time: 2-4h)	sclareol (0-41.8%), linalool acetate (11.3-37.6%), linalool (5.1-35.8%), α-terpineol (2.7-11.0%), germacrene D (4.2-5.2%)	Oxygenated monoterpenes: 21.9-88.8%<br>Diterpenes: 0-52.3%<br>Sesquiterpene hydrocarbons: 5.2-7.4%<br>Monoterpene hydrocarbons: tr-5.8%<br>Oxygenated sesquiterpenes: tr-2.3%<br>Alkanes: 0-3.9%	[48]
	Greece (cultivated)	linalyl acetate (21.41%), caryophyllene oxide (11.41%), trans-caryophyllene (11.32%), α-copaene (7.43%), germacrene D (5.90%), sclareol (5.31%)	n.d.	[49]
	Iran	sclareol (11.0%), germacrene D (9.8%), linalool (9.0%), α-terpineol (7.4%)	n.d.	[50]
	Iran (four accessions)	linalool (12.2-21.4%), α-terpineol (3.56-5.58%), linalyl acetate (13.1-52.6%), (E)-caryophyllene (3.08-21.3%), germacrene D (9.62-17.7%), germacrene A (0-5.77%), spathulenol (1.56-5.01%), caryophyllene oxide (0.31-8.53%)	n.d.	[18]
	Central Iran (flower; leaves)	germacrene D (20.88; 20.83%), β-caryophyllene (9.69; 17.24%), limonene (12.29; 2.12%), caryophyllene oxide (0; 10.42%), spathulenol (9.74; 9.98%), eudesm-7(11)-en-4-ol (9.95; 2.17%), bicyclogermacrene (3.37; 8.76%), α-copaene (0; 6.79%), β-longipinene (5.52; 0%), α-ylangene (5.02; 0%)	n.d.	[51]
	Italy	α-terpineol (47.4%), α-terpinyl acetate (22.1%), linalyl acetate (12.7%)	Alcohols: 50.9%<br>Esters: 38.2%<br>Hydrocarbons: 7.97%	[52]
	Southern Italy (cultivated; inflorescences, leaves)	linalool (n.d.-28.91%), linalyl acetate (n.d.-52.7%), germacrene D (3.92-68.85%), α-terpineol (n.d.-5.08%)	n.d.	[53]

	Central Italy	linalool (24.5%), linalyl acetate (20.9%), β-myrcene (8.4%), α-terpineol (9.8%), geranyl acetate (6.3%), (E)-β-ocimene (5.7%), caryophyllene oxide (5.3%)	n.d.	[54]
	Italy	linalyl acetate (59.3%), linalool (11.3%), germacrene D (10.5%)	Monoterpene hydrocarbons: 71.9%<br>Sesquiterpene hydrocarbons: 19.8%<br>Oxygenated monoterpenes: 6.4%<br>Oxygenated sesquiterpenes: 1.0%<br>Others: 0.8%	[55]
	Lebanon (Beirut; Taanayel)	linalool (38.07; 10.75%), linalyl acetate (35.28; 1.0%), α-terpineol (13.40; 6.45%), geraniol (5.67; 4.40%), germacrene D (2.0; 10.60%)	Oxygenated monoterpenes: 66.28; 66.04%<br>Monoterpene hydrocarbons: 7.92; 7.68%<br>Sesquiterpene hydrocarbons: 5.94; 5.70%<br>Oxygenated sesquiterpenes: 5.22; 4.98%<br>Others: 1.44; 0.96%	[56]
	Poland	camphene (23.36%), thujol (12.31%)	n.d.	[57]
	Serbia (cultivated)	linalyl acetate (43.5%), linalool (25.9%), α-terpineol (5.0%), germacrene D (5.0%)	Oxygenated monoterpenes: 82.2%<br>Sesquiterpene hydrocarbons: 10.9%<br>Monoterpene hydrocarbons: 2.3%<br>Oxygenated diterpenes: 1.6%<br>Oxygenated sesquiterpenes: 0.7%	[58]
	Serbia	linalyl acetate (97.7%)	Oxygenated monoterpenes and their acetyl derivatives: 99.7%	[24]
	Slovakia (cultivated/ flower; leaves)	linalool (18.9; 0.2%), linalyl acetate (13.7; 0%), α-terpineol (6.5; 0%), α-copaene (1.1; 6.0%), β-caryophyllene (2.1; 12.3%), germacrene D (5.0; 28.8%), bicyclogermacrene (0.7; 12.5%), spathulenol (0.6; 10.1%), caryophyllene oxide (0.8; 6.2%), sclareol (15.7; 0%)	n.d.	[59]
	Spain	linalool (32.97%), linalyl acetate (16.85%), germacrene D (7.57%), α-terpineol (5.63%)	n.d.	[25]

	Uruguay (cultivated)	linalool (7.9-22.5%), linalyl acetate (38.6-48.1%), germacrene D (8.2-19.8%)	Oxygenated monoterpenes: 52.7-75.2%<br>Sesquiterpene hydrocarbons: 17.4-30.5%<br>Monoterpene hydrocarbons: 4.0-4.5%<br>Oxygenated sesquiterpenes: 0.2-0.6%<br>Others: 1.1-2.7%	[15]
	Tajikistan	linalyl acetate (39.2%), linalool (12.5%), germacrene D (11.4%), α-terpineol (5.5%)	n.d.	[60]
	Turkey	caryophyllene oxide (24.1%), spathulenol (11.4%), sclareol (11.5%), 1H-naphtho(2,1,6)pyran (8.6%), β-caryophyllene (5.1%)	n.d.	[61]
	Turkey	spathulenol (19.0%), caryophyllene oxide (15.5%), linalyl acetate (11.3%), linalool (8.5%), naphtho(2,1,6)pyran (7.0%)	n.d.	[62]
	Turkey	heptacosane (9.46%), tert-hexadecanethiol (7.55%), cetyl alcohol (5.79%)	Hydrocarbons and derivatives: 31.27%<br>Monoterpene hydrocarbons: 4.58%<br>Oxygenated monoterpenes: 5.60%<br>Sesquiterpene hydrocarbons: 9.53%<br>Oxygenated sesquiterpenes: 21.0%<br>Diterpene alcohols: 6.28%<br>Fatty acids and derivatives: 5.09%<br>Phenolic compounds: 0.27%<br>Oxygenated triterpenes: 0.35%<br>Other compounds: 1.66%	[28]
	Southern Uzbekistan (cultivated and wild-growing / different plant parts)	linalool, linalyl acetate, α-terpineol	n.d.	[63]
	Uzbekistan	9-octadecenoic acid (6.9%), n-butyl octadecenoate (5.7%)	n.d.	[64]
***Eusphace* section / I-D clade**				
S. ringens	Greece	1,8-cineole (46.42-50.74%), α-pinene (10.64-12.85%), bornyl acetate (4.53-6.54%), β-pinene (4.34-5.64%)	Monoterpene alcohols: 57.03-64.94%<br>Monoterpene hydrocarbons: 25.06-27.57%	[65]

	(two samples of different collection years)			
	Greece	*α*-pinene (28.1%), β-pinene (12.2%), 1,8-cineole (13.0%), camphene (6.9%), borneol (6.6%)	Monoterpene hydrocarbons: 57.4%<br>Oxygenated monoterpenes: 33.5%<br>Sesquiterpene hydrocarbons: 5.7%<br>Oxygenated sesquiterpenes: 3.4%	[66]
	Bulgaria (leaves; flowers)	camphor (17.2; 18.8%), borneol (7.2; 8.7%), β-pinene (6.0; 4.0%), camphene (1.1; 5.0%)	Monoterpenes: 64.3-68.4%<br>Sesquiterpenes: 28.0-32.3%<br>Cycloaliphatic compounds: 0.2%<br>Aromatic compounds: 3.1-3.3%	[67]
	North Macedonia	1,8-cineole (31.99%), camphene (17.06%), borneol (11.94%), *α*-pinene (11.52%)	Oxygenated monoterpenes: 56.89%<br>Monoterpene hydrocarbons: 36.74%<br>Aromatic hydrocarbons: 2.96%<br>Sesquiterpene hydrocarbons: 2.16%<br>Aliphatic hydrocarbons: 0.73%<br>Oxygenated sesquiterpenes: 0.16%	[68]
***Hemisphace* section / I-B clade**				
S. verticillata	Czech Republic<br>(seven locations)	caryophyllene (20.18-65.25%), humulene (15.56-26.59%), β-pinene (0.94-28.98%), limonene (0.63-13.92%), myrcene (0.56-6.09%), *α*-pinene (1.23-5.16%), linalool (1.60-5.04%)	n.d.	[78]
	Greece	β-pinene (30.7%), p-cymene (23.0%), isopropyl ester of lauric acid (16.8%), *α*-pinene (7.6%), (E)-nerolidol (5.2%)	Monoterpenes: 64.5%	[39]
	Italy<br>(three sampling periods)	germacrene D (39.5-40.7%), bicyclogermacrene (11.5-14.8%), β-caryophyllene (7.3-11.9%), spathulenol (3.1-6.6%), *α*-humulene (2.7-5.9%)	Sesquiterpene hydrocarbons: 72.3-79.6%<br>Monoterpene hydrocarbons: 9.9-18.2%<br>Oxygenated sesquiterpenes: 6.3-7.0%<br>Oxygenated monoterpenes: 0.4-0.8%<br>Sulphured sesquiterpenes: 0-0.3%<br>Non-terpene derivatives: tr-1.2%	[77]

	Iran	β-caryophyllene (24.7%), γ-muurolene (22.8%), limonene (8.9%), α-humulene (7.8%), β-pinene (5.1%)	n.d.	[69]
	Iran (cultivated; wild-growing populations)	(E)-caryophyllene (17.813; 14.706%), α-gurjunene (0; 12.825%), β-phellandrene (14.236; 6.614%), α-humulene (10.162; 7.664%), α-pinene (5.735%), germacrene D (5.179; 8.684%), β-pinene (0; 6.541%), bicyclogermacrene (0; 6.384%)	n.d.	[70]
	Iran	trans-caryophyllene (24.40%), β-phellandrene (9.08%), α-humulene (8.61%), bicyclogermacrene (6.32%), spathulenol (5.89%), β-pinene (5.0%)	Sesquiterpene hydrocarbons: 52.92%<br>Monoterpene hydrocarbons: 34.10%<br>Sesquiterpenes oxygenated: 8.92%<br>Unknown: 2.33%<br>Diterpenoids: 0.84%<br>Monoterpenes oxygenated: 0.73%<br>Nonterpenes: 0.16%	[71]
	Iran (three locations)	(E)-caryophyllene (17-41%), bicyclogermacrene (1.73-21%), α-humulene (5.42-14%), germacrene D (3.47-13%), spathulenol (0-17%), caryophyllene oxide (0-10%), α-gurjunene (0-5.66%)	n.d.	[18]
	Iran	1,8-cineole (38.26%), camphor (22.98%), bicycloheptan (5.52%)	n.d.	[72]
	Iran (different locations/ flowering stages)	(E)-caryophyllene (6.7-41.0%), α-humulene (2.9-15.9%), germacrene D (0.4-13.9%), bicyclogermacrene (1.5-10.6%), (E,E-α)-farnesene (t-29.1%), germacrene B (0.9-6.2%), spathulenol (0.2-15.3%),	n.d.	[73]
	Poland	α-pinene (10.72%), camphor (5.23%), limonene (5.85%)	n.d.	[57]
	Serbia (three populations)	germacrene D (0-48.0%), (E)-caryophyllene (10.2-19.0%), α-humulene (4.8-10.2%), bicyclogermacrene	n.d.	[76]

		(5.3-16.7%), spathulenol (3.5-7.2%), α-cadinol (t-10.4%), β-phellandrene (t-8.6%), β-cubebene (0-8.6%), eicosane (0-8.5%), (Z)-β-ocimene (0-6.0%), (E)-β-ocimene (0-7.5%), δ-cadinene (t-6.0%), eudesma-4(15),7-dien-1β-ol (0-6.0%)		
	Serbia (three locations)	β-phellandrene (43.9-70.4%), (E)-β-ocimene (1.0-12.2%), (Z)-β-ocimene (1.7-10.3%), myrcene (6.0-6.6%), α-pinene (1.9-21.1%), sabinene (1.7-5.5%)	Monoterpene hydrocarbons: 89.3-98.8%<br>Sesquiterpene hydrocarbons: 0.3-9.3%<br>Other: 0.5-0.9%	[24]
	Former Yugoslavia	β-caryophyllene (13.3%), γ-muurolene (10.3%), trans-chrysanthenol (6.1%), α-humulene (5.4%), 1,8-cineole+ β-phellandrene (5.0%)	n.d.	[30]
***Plethiosphace* section / I-C clade**				
S. amplexicaulis	Poland	camphene (24.0%), β-chamigrene (8.5%), thujol (7.63%)	n.d.	[57]
	Serbia	germacrene D (14.8%), viridiflorol (10.6%), caryophyllene oxide (10.5%), β-caryophyllene (9.4%), eudesma-4(15),7-dien-1β-ol (5.2%)	Sesquiterpene hydrocarbons: 44.5%<br>Oxygenated sesquiterpenes: 36.6%<br>Monoterpenes: 1.6%	[79]
	Serbia	germacrene D (21.0%), caryophyllene oxide (15.1%), (E)-caryophyllene (9.2%), α-cadinol (6.7%), germacra-4(15),5,10(14)-trien-1α-ol (5.4%), trans-phytol (5.1%)	Oxygenated sesquiterpenes: 46.5%<br>Sesquiterpene hydrocarbons: 43.0%<br>Oxygenated diterpenes: 5.1%<br>Oxygenated monoterpenes: 1.1%<br>Monoterpene hydrocarbons: 0.4%	[80]
	Serbia	o-cymene (21.1%), 1-octen-3-ol (18.9%), sabinene (14.2%), limonene (11.2%), α-thujene (10.2%)	Monoterpene hydrocarbons: 57.0%<br>Other: 42.6%	[24]
S. pratensis	Serbia	caryophyllene oxide (35.1%), Z-caryophyllene (11.4%)	Oxygenated sesquiterpenes: 35.1%<br>Sesquiterpene hydrocarbons: 21.9%<br>Aliphatic components: 10.8%<br>Oxygenated monoterpenes: 7.2%<br>Monoterpene hydrocarbons: 0.2%	[82]

	Serbia	(E)-caryophyllene (26.4%), (Z)-β-farnesene (6.0%), β-cubebene (5.6%), epi-bicyclo sesquiphellandrene (5.6%)	Sesquiterpene hydrocarbons: 53.7%<br>Aliphatic components: 15.7%<br>Oxygenated sesquiterpenes: 1.4%<br>Oxygenated monoterpenes: 1.1%<br>Monoterpene hydrocarbons: 0.3%	[82]
	Poland	camphene (17.42%), thujol (9.11%)	n.d.	[57]
S. virgata	Iran	β-caryophyllene (46.6%), germacrene-B (13.9%), β-caryophyllene epoxide (13.2%), spathulenol (6.4%), germacrene-D (5.7%)	Sesquiterpenes: 90%	[83]
	Iran	caryophyllene oxide (34.4%), spathulenol (25.6%), 1-docosanol (11.7%), n-tetradecanol (9.3%), geranyl ac etone (5.6%)	n.d.	[84]
	Iran	caryophyllene oxide (61.5%)	n.d.	[85]
	Iran<br>(leaves; steam; aerial parts)	β-caryophyllene (35.2;7.6;23.1%), (Z)-β-farnesene (10.1;2.4;12.3%), sabinene (3.6;2.2;18.2%), caryophyllene oxide (6.1;0.5;2.3%), hexadecenoic acid (0.5;56.0;5.0%), α-pinene (5.7;2.5;3.2%)	Sesquiterpene hydrocarbons: 50.7;11.0;41.2%<br>Oxygenated sesquiterpenes: 11.8;4.2;5.4%<br>Fatty acids: 0.5; 59.2;5.3%<br>Monoterpene hydrocarbons: t;t;4.2%<br>Others:11.2;3.4;4.1%	[86]
	Iran<br>(pre-flowering and full-flowering stage)	β-caryophyllene (24.58-42.54 %), caryophyllene oxide (10.25-19.88 %), sabinene (8.64-19.58 %), 1-octen -3-ol (7.54-8.59 %), terpinene-4-ol (4.25-6.64 %), α-thujene (3.74-6.46 %)	n.d.	[87]
	Iran	(E)-caryophyllene (30.0%), δ-cadinene (16.0%), caryophyllene oxide (10.0%), (E)-β-farnesene (8.6%), γ-gurjunene (6.54%), γ-cadinene (6.17%), linalool acetate (5.2%)	n.d.	[18]
	Iran	caryophyllene oxide (30.23%), β-caryophyllene (22.63%), sabinene	n.d.	[88]

		(11.82%), 1-octan-3-ol (6.64%), thujene (6.28%), terpinene-4-ol (5.25%)		
	Iran	(E)-caryophyllene (34.2-37.7 %), caryophyllene oxide (26.2-29.4 %), (E)-β-farnesene (8.5-9.9 %), δ-cadinene (5.4-6.7%)	n.d.	[89]
	Iran	pentacosane (20.09%), caryophyllene oxide (6.90%), phytol (6.83%), spathulenol (6.09%), nonacosane (5.15%)	n.d.	[90]
	Turkey	caryophyllene oxide (28.3%), thymol (19.8%), spathulenol (13.2%), trans-caryophyllene (9.1%)	n.d.	[91]
	Turkey	borneol (23.41%), palmitic acid (7.93%), trans-pinocarvyl acetate (5.06%)	Oxygenated monoterpenes:42.30%<br>Hydrocarbons and derivatives: 11.82%<br>Monoterpene hydrocarbons: 2.49%<br>Sesquiterpene hydrocarbons: 1.33%<br>Oxygenated sesquiterpenes: 7.23%<br>Fatty acids and derivatives: 11.90%<br>Phenolic compounds: 0.11%<br>Oxygenated triterpenes: 0.29%<br>Others: 0.29%	[28]
	Turkey	1,8-cineole (20.3 %), α-copaene (18.6 %), germacrene D (17.6 %)	n.d.	[92]

n.d.= not determined

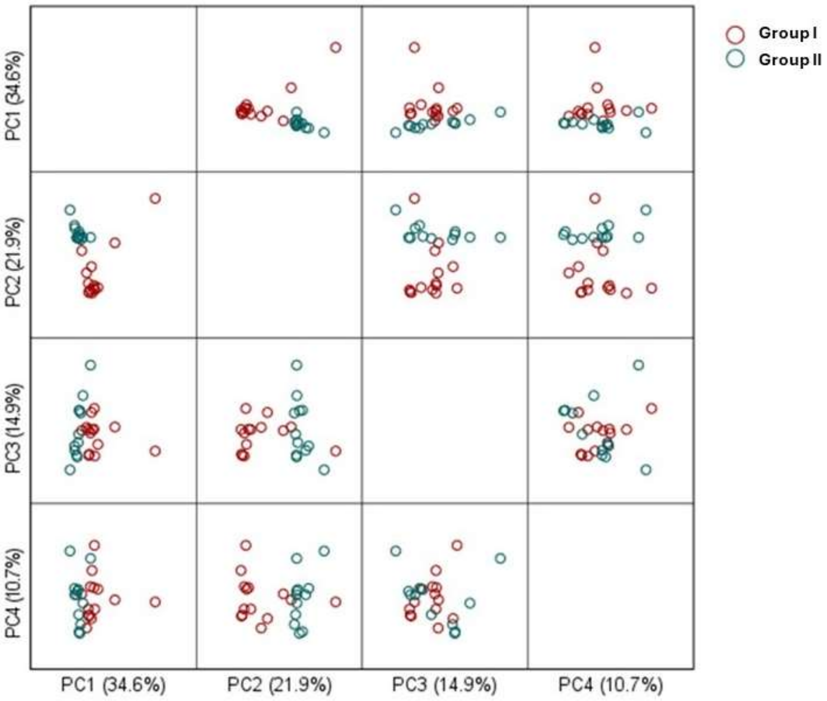

Figure S1. Principal Component Analysis of the major chemical classes by *Salvia* groups as defined in HCA.

The PCA elucidated 82% of the data variability. PC1 explained 34.6% and showed positive correlations with Sesquiterpene Hydrocarbons (SH, r= 0.30), Oxygenated Sesquiterpenes (OS, r= 0.16), Hydrocarbons-Alcohols (HAlc, r= 0.90), Hydrocarbons-Aldehydes (Hald, r= 0.91), Hydrocarbons-Ketones (HK, r= 0.86), Hydrocarbons-Alkanes (HAlk, r= 0.85) and Others (OT, r= 0.14), as well as negative correlations with Monoterpene Hydrocarbons (MH, r = -0.05), Oxygenated Monoterpenes (OM, r= -0.52), Diterpenes (D, r= -0.27) and Oxygenated diterpenes (OD, r= -0.46). The second component explained 21.9% of the total variability and presented positive correlations with Monoterpene Hydrocarbons (MH, r= 0.30), Oxygenated Monoterpenes (OM, r= 0.76), Diterpenes (D, r= 0.34), Oxygenated diterpenes (OD, r= 0.51), Hydrocarbons-Alcohols (HAlc, r= 0.28), Hydrocarbons-Aldehydes (Hald, r= 0.35), Hydrocarbons-Ketones (HK, r= 0.17), Hydrocarbons-Alkanes (HAlk, r= 0.38) and Others (OT, r= 0.07), while negative correlations with Sesquiterpene Hydrocarbons (SH, r= -0.69) and Oxygenated Sesquiterpenes (OS, r= -0.71). The third component explained 14.9% and displayed positive correlations with Monoterpene Hydrocarbons (MH, r= 0.77), Oxygenated Sesquiterpenes (OS, r= 0.13), Hydrocarbons-Ketones (HK, r= 0.21) and Others (OT, r= 0.67), as well as negative correlations with Oxygenated Monoterpenes (OM, r= -0.03), Sesquiterpene Hydrocarbons (SH, r= -0.40), Diterpenes (D, r= -0.36), Oxygenated diterpenes (OD, r= -0.40), Hydrocarbons-Alcohols (HAlc, r= -0.21), Hydrocarbons-Aldehydes (Hald, r= -0.13) and Hydrocarbons-Alkanes (HAlk, r= -0.16). The fourth component explained the 10.7% of the total variability and is positively correlated with Oxygenated Sesquiterpenes (OS, r= 0.49), Diterpenes (D, r= 0.42), Oxygenated diterpenes (OD, r= 0.40), Hydrocarbons-Ketones (HK, r= 0.24) and Others (OT, r= 0.57), while negatively correlated with Monoterpene Hydrocarbons (MH, r= -0.39), Oxygenated Monoterpenes (OM, r= -0.12), Sesquiterpene Hydrocarbons (SH, r= -0.22), Hydrocarbons-Alcohols (HAlc, r= -0.05), Hydrocarbons-Aldehydes (Hald, r= -0.03) and Hydrocarbons-Alkanes (HAlk, r= -0.01). The PCA analysis formed four distinct components (two groups; Group I and II, Figure S1).
